# Mitochondrial Genome Characterization of Six Spiny Crawler Mayflies and Comparative Analysis Within Ephemerellidae (Ephemeroptera: Pannota)

**DOI:** 10.1002/ece3.72935

**Published:** 2026-01-08

**Authors:** Chao Xue, Zhenxing Ma, Dongkai Liu, Ran Li, Xianfeng Yi

**Affiliations:** ^1^ School of Life Sciences Qufu Normal University Qufu China; ^2^ School of Food Science and Biology Engineering Wuhu Institute of Technology Wuhu China

**Keywords:** Ephemerellidae, Ephemeroptera, mayfly, mitochondrial genome, phylogenetic analysis

## Abstract

Mitogenomes provide important molecular information for resolving evolutionary relationships in mayflies. However, both the evolutionary patterns of mitogenomes and the phylogenetic structure of Ephemerellidae remain insufficiently understood. In this study, we sequenced six complete mitogenomes representing five ephemerellid genera and performed comprehensive comparative analyses that included all currently available ephemerellid mitogenomes. Our results reveal that *trnI*‐associated inversion and translocation are characteristic features of Ephemerellidae mitogenomes, likely generated through tandem duplication followed by random loss during mitochondrial evolution. All species exhibited strong A + T bias and distinct compositional asymmetry, with codon usage heavily skewed toward A/T at third codon positions. Nucleotide diversity and evolutionary rate analyses indicated that *ND6* and *ND2* were the most variable protein‐coding genes, whereas *COX1* was the most conserved among the 13 protein‐coding genes. Phylogenetic analyses based on 13 PCGs and two rRNAs using Bayesian inference (BI) and Maximum likelihood (ML) consistently supported the monophyly of *Torleya*, *Cincticostella*, and *Serratella*, and recovered stable relationships among the major lineages within Ephemerellidae. Overall, the six newly sequenced mitogenomes enrich the mitochondrial genomic resources for Ephemerellidae and provide valuable insights into mitogenome evolution and phylogenetic relationships in this family.

## Graphical abstract

Ephemerellidae is a diverse family of mayflies widely distributed in the Holarctic and Oriental regions, comprising more than 300 described species. However, mitochondrial genomic data for this family remain scarce, limiting our understanding of its evolutionary history. In this study, we sequenced and annotated six complete mitogenomes representing five genera of Ephemerellidae. The distinct patterns of tRNA gene rearrangement and duplication revealed here provide new insights into the evolution and diversity of Ephemerellidae and offer valuable molecular resources for phylogenetic and taxonomic studies within Ephemeroptera.

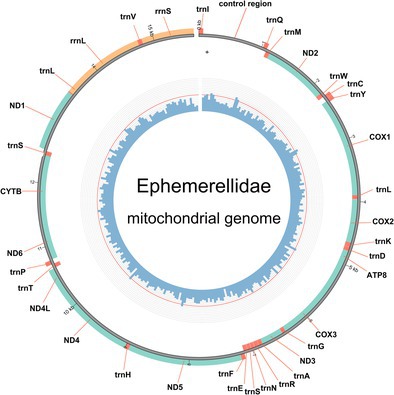

## 1 Introduction

The order Ephemeroptera (mayflies) represents one of the most ancient lineages of winged insects, with fossil evidence tracing back to the Carboniferous period, approximately 300 million years ago (Bauernfeind and Soldán 2012). Comprising about 42 families and nearly 4000 species, mayflies are globally distributed and inhabit freshwater ecosystems on every continent except Antarctica (Jacobus et al. 2021). They exhibit a distinctive prometabolous metamorphosis, being the only insects that molt once after wing formation to achieve sexual maturity (Sartori and Brittain 2015; Kamsoi et al. 2021). The nymphal stage dominates their life cycle and plays vital roles in nutrient cycling and energy flow within aquatic food webs. Depending on species and habitat, nymphs function as scrapers, collectors, shredders, or predators (Baptista et al. 2006). Their gill‐breathing habit, close association with benthic substrates, and low tolerance to pollution render them excellent bioindicators of freshwater ecosystem health (Rodriguez et al. 2018; Benhadji et al. 2025).

Among the families of Ephemeroptera, Ephemerellidae (commonly known as spiny crawler mayflies) is one of the most species‐rich and widely distributed groups, comprising 18 genera and more than 300 described species (Barber‐James et al. 2008; McCafferty and Wang 2000). Members of this family are distributed mainly across the Palearctic and Oriental regions, extending from tropical to subarctic zones. Their nymphs inhabit a broad range of freshwater environments, from fast‐flowing streams to lake margins, and are particularly abundant in high‐latitude running waters (Allen and Edmunds 1963; Yoon et al. 1985). Ecologically, ephemerellid nymphs are highly sensitive to environmental variation and are often used as bioindicators for assessing freshwater ecosystem integrity (Buchwalter et al. 2007; Jacobus and McCafferty 2008). They also serve as an important food source for insectivorous fishes and birds (Feck and Hall 2004). Morphologically, Ephemerellidae is characterized by dorsoventrally flattened nymphs bearing dorsal tubercles, reduced gills on posterior abdominal segments, and distinctive thoracic and caudal structures (Kluge 2004). However, despite extensive morphological research, phylogenetic relationships within the family and its affinities with related families such as Vietnamellidae and Teloganellidae remain controversial, largely due to morphological convergence and limited molecular evidence (Ogden and Whiting 2005).

Insect mitochondrial genomes (mitogenomes) are typically circular double‐stranded DNA molecules of 14–20 kb, comprising 37 genes: 13 protein‐coding genes (PCGs), 22 transfer RNAs (tRNAs), two ribosomal RNAs (rRNAs), and a noncoding control region (CR) (Boore 1999; Cameron 2014). Their relatively small size, conserved gene content, maternal inheritance, absence of recombination, and generally rapid evolutionary rate make them highly informative molecular markers for evolutionary and phylogenetic studies (Sun et al. 2025; Korkmaz et al. 2017; Liu et al. 2025). The advent of high‐throughput sequencing technologies has facilitated the efficient acquisition of complete mitogenomes, providing robust datasets for comparative genomic and phylogenetic analyses. Compared with single‐gene or short‐fragment approaches, complete mitogenomes offer higher resolution in reconstructing deep phylogenetic relationships by integrating both sequence and structural features, such as nucleotide composition bias, codon usage patterns, AT‐ and GC‐skews, gene rearrangement patterns, and the secondary structures of tRNAs and rRNAs (Ge et al. 2023). Consequently, mitogenome‐based analyses have become powerful tools for exploring evolutionary dynamics within and among insect lineages.

Recent mitogenomic studies have greatly advanced our understanding of the phylogeny and evolution of Ephemeroptera. Although most mayfly mitogenomes retain the ancestral insect gene order, several families exhibit distinct gene rearrangements, particularly among tRNA genes (Li et al. 2014). For instance, rearrangements involving *trnM*, *trnI*, and *trnQ* have been frequently reported in Heptageniidae and Baetidae (Li et al. 2021). Within Ephemerellidae, several studies have revealed characteristic gene rearrangements and unique structural features. Xu et al. (2020) analyzed six mitogenomes from *Ephemerella*, *Serratella*, and *Torleya* and identified inversions and duplications of *trnI*, with multiple copies detected in *Torleya*. Li et al. (2020) further reported a novel rearrangement pattern (*trnI*–CR–*trnQ*–*trnM*) in *Torleya mikhaili* and *Cincticostella fusca*, likely resulting from tandem duplication and random loss (TDRL) events (Li et al. 2020). These findings suggest that Ephemerellidae mitogenomes are evolutionarily dynamic and contain important phylogenetic signals. However, despite these advances, Ephemerellidae remains underrepresented in mitochondrial datasets compared with families such as Heptageniidae. Limited taxon sampling has constrained our understanding of genome architecture evolution, codon bias, compositional skew, substitution rates, and phylogenetic structure within the family. Expanding taxonomic coverage to include multiple genera and geographic populations is therefore essential for accurately reconstructing evolutionary relationships and testing competing phylogenetic hypotheses within Ephemerellidae (Xu et al. 2020; Li et al. 2020).

In this study, we sequenced and assembled the complete mitogenomes of six Ephemerellidae species collected from different regions of China, representing five genera within the family. Together with all available ephemerellid mitogenomes retrieved from GenBank, we performed comprehensive comparative genomic and phylogenetic analyses, examining genome organization, base composition, codon usage, and evolutionary rates of protein‐coding genes. Gene rearrangement patterns were characterized to assess their evolutionary implications, and phylogenetic relationships were reconstructed based on 13 PCGs and two rRNAs to reevaluate intergeneric relationships and test the monophyly of major lineages. By integrating newly generated and published data, this study provides the most extensive mitogenomic dataset for Ephemerellidae to date, offering new insights into mitochondrial genome evolution, gene rearrangement mechanisms, and phylogenetic relationships within this ecologically and taxonomically important family, and contributing valuable molecular resources for future evolutionary and biodiversity research on Ephemeroptera.

## 2 Materials and Methods

### 2.1 Sample Collection

Nymphal specimens of six Ephemerellidae species representing five genera were collected from various regions of China. Specifically, *Cincticostella femorata*, *Cincticostella gosei*, and *Uracanthella punctisetae* were collected from Hangzhou, Zhejiang Province; *Drunella ishiyamana* was collected from Aba Autonomous Prefecture, Sichuan Province; *Teloganopsis jinghongensis* was collected from Tengchong, Yunnan Province; and *Torleya nepalica* was collected from Luoyang, Henan Province. Specimens were morphologically identified by Dr. Changfa Zhou using available taxonomic keys.

All specimens were preserved in 100% ethanol in the field and subsequently stored at −20°C in the laboratory until DNA extraction. Morphological identification was performed based on external features and male genitalia using established taxonomic keys. Voucher specimens were deposited in the College of Life Sciences, Nanjing Normal University, China.

### 2.2 DNA Extraction and Sequencing

Total genomic DNA was extracted from thoracic muscle or leg tissues using the Tissue and Blood Genomic DNA Extraction Kit (Qiagen, Germany) following the manufacturer's instructions. DNA quality and concentration were assessed using 1% agarose gel electrophoresis and a NanoDrop 2000 spectrophotometer (Thermo Fisher Scientific, USA). High‐quality DNA was used for library construction and sequencing by Biozeron Biotechnology Co. Ltd. (Shanghai, China). Sequencing libraries were prepared with the TruSeq Nano DNA HT Sample Preparation Kit (Illumina, USA). Paired‐end sequencing (PE150) was performed on the Illumina HiSeq 2500 platform, generating approximately 3–4 Gb of clean data per sample.

### 2.3 Mitogenome Assembly and Annotation

Raw reads were filtered using fastp v0.23.2 to remove adapters and low‐quality sequences (Chen et al. 2018), and sequence quality was verified with FastQC v0.11.9 (Brown et al. 2017). Clean reads were *de novo* assembled in Geneious v11.1.5 using closely related Ephemerellidae mitogenomes as references (Kearse et al. 2012). Preliminary annotations were generated in MitoZ v2.4 and manually refined in Geneious v11.1.5 for accuracy (Meng et al. 2019). The positions of 13 PCGs were confirmed by comparison with homologous sequences from other Ephemerellidae species to verify start and stop codons. Transfer RNA (tRNA) genes were predicted with ARWEN (Laslett and Canbäck 2008) and tRNAscan‐SE (Chan et al. 2021), and ribosomal RNA (rRNA) genes were identified using MITOS (Bernt et al. 2013). The control region, intergenic spacers, and overlapping regions were determined and validated in MEGA X (Kumar et al. 2018).

### 2.4 Sequence Analysis

Base composition and codon usage for all 13 PCGs were calculated using MEGA X and PhyloSuite v1.2.1 (Zhang et al. 2020). Relative synonymous codon usage (RSCU) values were computed in PhyloSuite. Strand compositional asymmetry was evaluated using AT‐skew = (A − T)/(A + T) and GC‐skew = (G − C)/(G + C). The nonsynonymous (Ka) and synonymous (Ks) substitution rates and their ratios (Ka/Ks) were calculated for all PCGs using DnaSP v6.0 (Rozas et al. 2017). Repeat motifs within the control region were identified with Tandem Repeats Finder (Benson 1999).

### 2.5 Alignment and Phylogenetic Analysis

Phylogenetic analyses were performed using a total of 12 Ephemerellidae mitogenomes, including six newly sequenced species and six previously published sequences from GenBank (Table 1). *Choroterpides apiculata* (Leptophlebiidae) and *Vietnamella dabieshanensis* (Vietnamellidae) were selected as the outgroups. The nucleotide sequences of all 13 PCGs and two rRNAs were used for analysis. Each PCG was aligned separately using codon‐based multiple alignments in TranslatorX under the MAFFT algorithm with the L‐INS‐i strategy and default parameters (Katoh and Standley 2013). The two rRNAs were aligned independently using MAFFT v7.205 with the G‐INS‐i strategy. Conserved regions were identified with Gblocks v0.91b using default settings to remove ambiguously aligned sites.

**TABLE 1:** Species information used for comparative and phylogenetic analyses.

Genus	Species	Size (bp)	GenBank
*Cincticostella*	*Cincticostella fusca*	15,135	MT535767
	*Cincticostella femorata*	15,594	PX508693
	*Cincticostella gosei*	15,416	PX508691
*Drunella*	*Drunella ishiyamana*	16,485	PX508690
*Serratella*	*Serratella ignita*	14,772	MT628582
	*Serratella zapekinae*	15,703	MT274130
*Teloganopsis*	*Teloganopsis jinghongensis*	15,626	PX508687
*Torleya*	*Torleya grandiforceps*	15,330	MT274131
	*Torleya mikhaili*	15,042	MT535766
	*Torleya tumiforceps*	15,599	MT274132
	*Torleya nepalica*	15,354	PX508688
*Uracanthella*	*Uracanthella punctisetae*	15,435	PX508692
Outgroup	*Choroterpides apiculata*	15,199	MN807287
	*Vietnamella dabieshanensis*	15,761	HM067837

The optimal partitioning scheme and best‐fit nucleotide substitution models for each dataset were determined using PartitionFinder 2 implemented in PhyloSuite under a greedy search algorithm with linked branch lengths and Bayesian Information Criterion (BIC) (Lanfear et al. 2017). Phylogenetic trees were reconstructed using both Maximum likelihood (ML) and Bayesian inference (BI) methods. ML analyses were conducted in IQ‐TREE v1.7 with 5000 ultrafast bootstrap replicates (Nguyen et al. 2015). BI analyses were performed in MrBayes v3.2.6 with two independent Markov Chain Monte Carlo (MCMC) runs of 1000,000 generations, sampling every 1000 generations and discarding the first 25% as burn‐in (Ronquist et al. 2012). The resulting phylogenetic trees were visualized and edited using FigTree v1.4.2 (Rambaut 2019).

## 3 Results

### 3.1 Genomic Organization and Gene Arrangement

We successfully sequenced and annotated the complete mitochondrial genomes of six ephemerellid species belonging to five genera: *D. ishiyamana*, *T. jinghongensis*, *T. nepalica*, *U. punctisetae*, and two *Cincticostella* species ( *C. femorata* and *C. gosei*). The total lengths of these mitogenomes ranged from 15,354 to 16,485 bp (Tables S1–, S6), which lies within the typical range reported for mayflies (14.5–16.6 kb) (Li et al. 2014; Gao et al. 2018). Substantial size variation was observed among ephemerellid species. For instance, *T. mikhaili* contains the smallest known ephemerellid mitogenome (15,042 bp), whereas *D. ishiyamana* possesses the largest (16,485 bp, Table 1). This variation in genome size primarily resulted from differences in the lengths of the control region and intergenic spacers.

**FIGURE 1:**
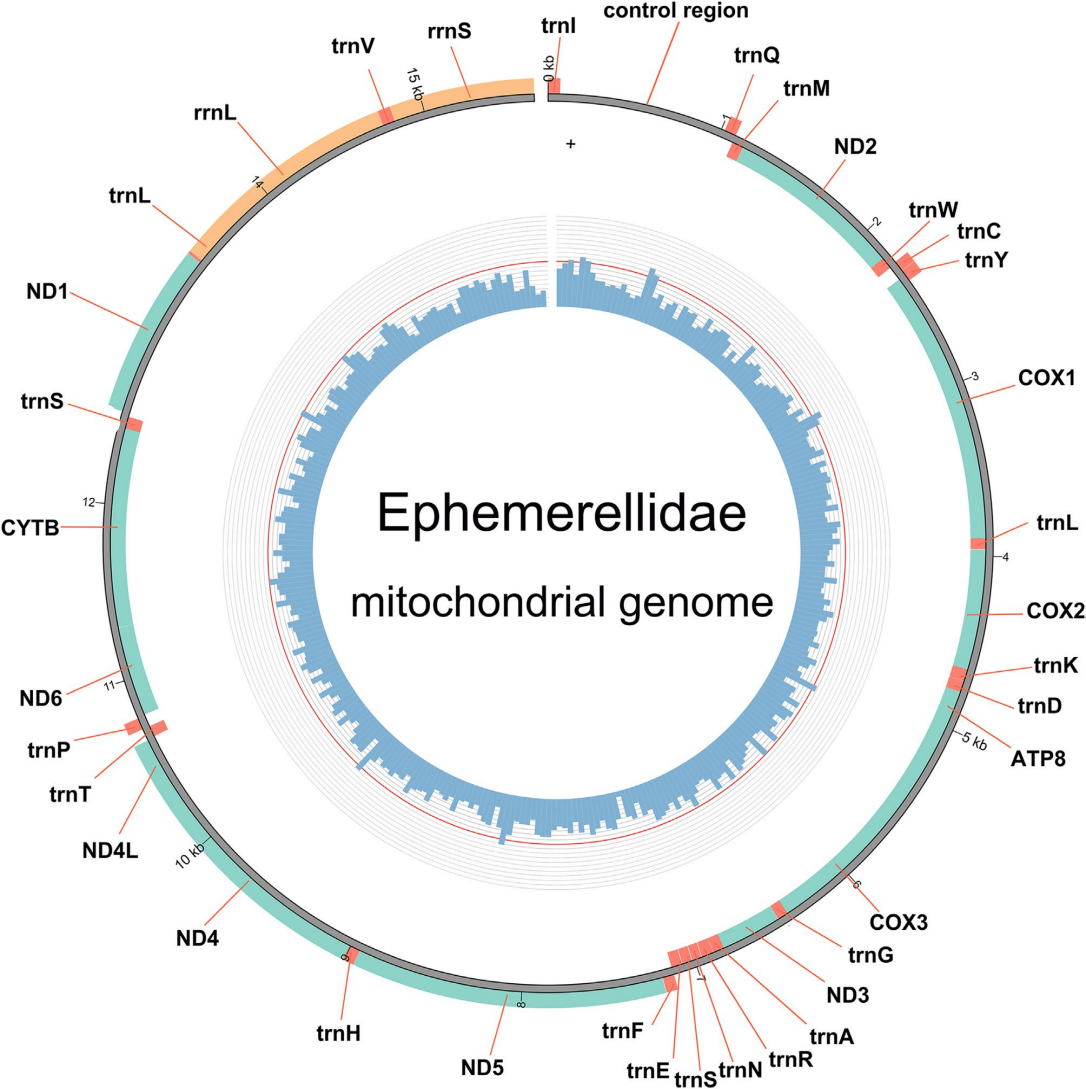
Mitochondrial map of Ephemerellidae. Genes transcribed clockwise on the inside and counterclockwise on the outside.

All six mitogenomes displayed the typical circular, double‐stranded organization and contained the standard set of 37 genes common to insect mitogenomes: 13 protein‐coding genes (PCGs), 22 transfer RNAs (tRNAs), two ribosomal RNAs (rRNAs), and one non‐coding control region (CR) (Figure 1). *T. nepalica* contains four additional copies of *trnI*, indicating lineage‐specific gene duplication events (Table S5). Similar *trnI* duplications have also been reported in other *Torleya* species (Xu et al. 2020).

The gene arrangement patterns of all six newly sequenced mitogenomes differed from the ancestral insect mitochondrial gene order (Clary and Wolstenholme 1985). In particular, *trnI* underwent both inversion and translocation, shifting from its ancestral position between the control region (CR) and *trnQ* to a location between rrnS and the CR. This rearrangement pattern is consistent with previous reports for *Ephemerella*, *Serratella*, and *Torleya* (Xu et al. 2020; Li et al. 2020).

### 3.2 Nucleotide Composition and Compositional Bias

**FIGURE 2:**
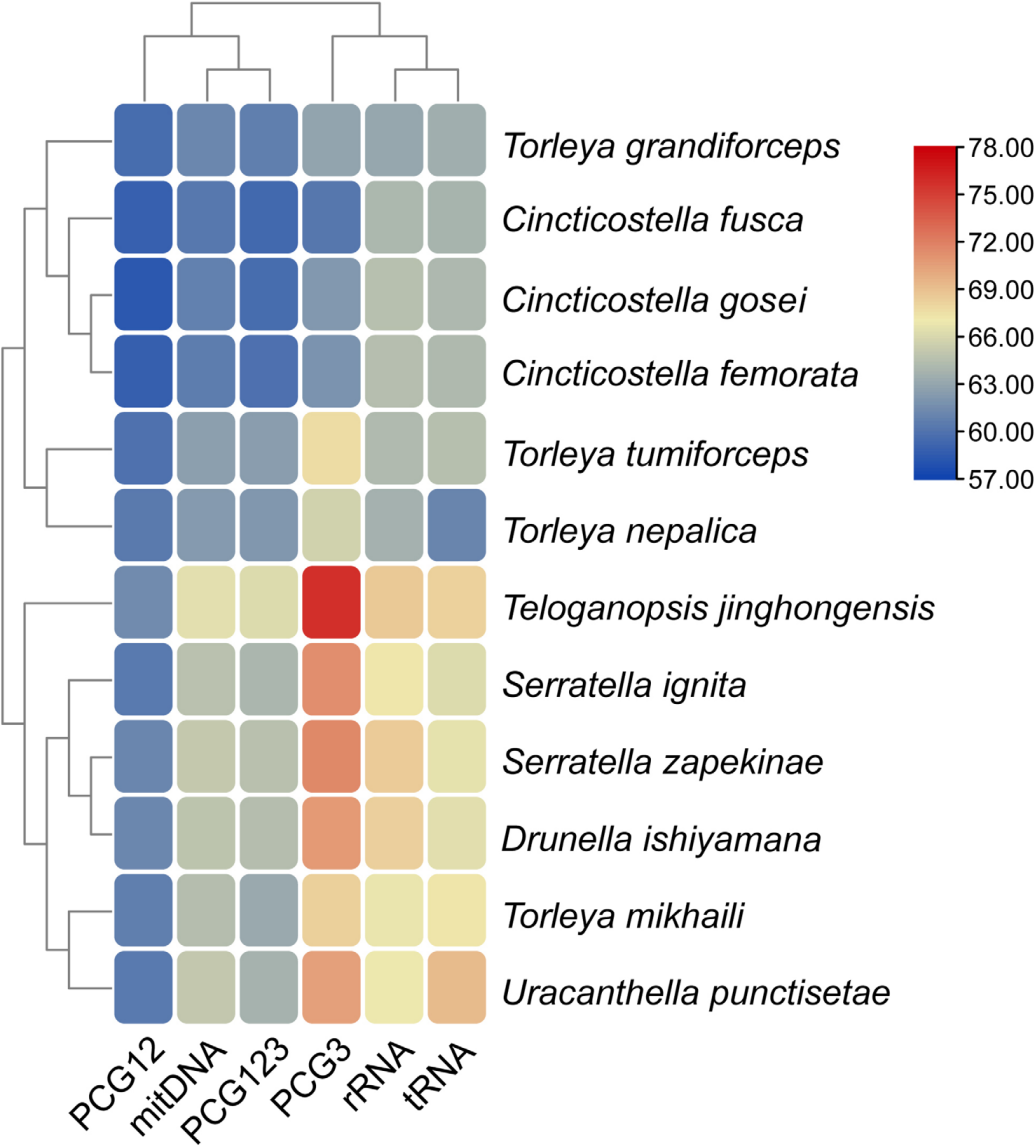
Nucleotide composition of various datasets of 12 Ephemerellidae mitogenomes. Hierarchical clustering of mayfly species (*y*‐axis) based on A + T content. PCG12: all PCGs with only the first and second codon positions, PCG3: all PCGs with the third codon positions, PCG123: all PCGs with three codon positions.

The nucleotide compositions of the six Ephemerellidae mitogenomes are summarized in Table S7. All species displayed a strong bias toward adenine (A) and thymine (T). The overall A + T content ranged from 60.58% in *C. femorata* to 66.53% in *T. jinghongensis*, consistent with values reported for other mayfly families (Li et al. 2025). Among the newly sequenced species, *D. ishiyamana* (64.82%) and *U. punctisetae* (65.05%) exhibited relatively high A + T contents, whereas *C. femorata* and *C. gosei* (60.83%) had the lowest (Figure 2).

At the regional level, the A + T content of PCGs ranged from 59.85% ( *C. femorata* ) to 66.24% (*T. jinghongensis*) (Figure 2). The rRNAs were even more A + T‐rich (63.70%–68.59%), whereas tRNAs displayed slightly lower values (61.08%–69.26%).

**FIGURE 3:**
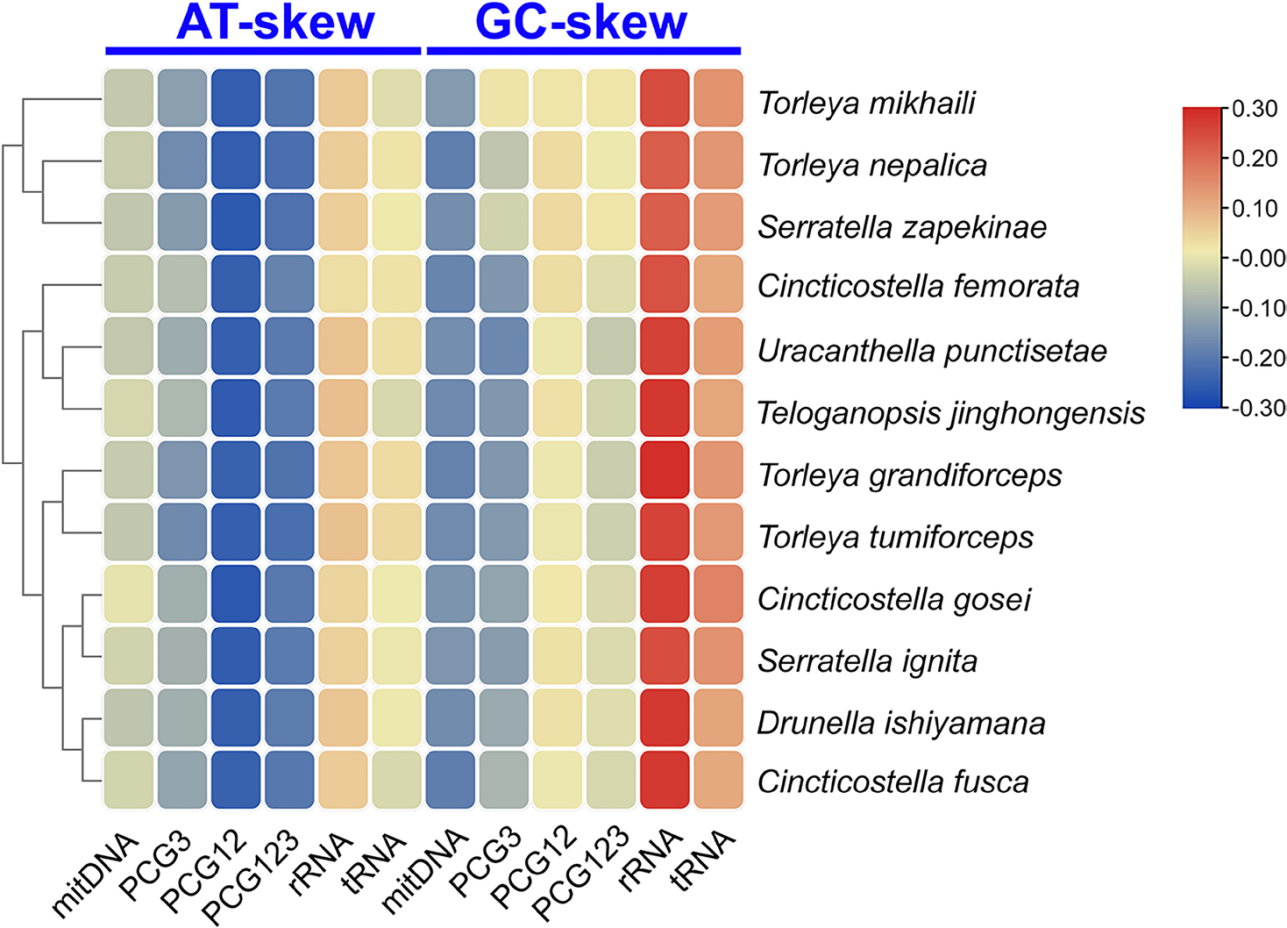
Nucleotide composition of various datasets of 12 Ephemerellidae mitogenomes. Hierarchical clustering of mayfly species (*y*‐axis) based on the AT‐skew and GC‐skew.

Nucleotide‐skew analysis revealed strand asymmetry. AT‐skew values for complete mitogenomes ranged from −0.0552 in *D. ishiyamana* to 0.0013 in *C. gosei* (Table S8). All PCGs exhibited negative AT‐skews (−0.1796 to −0.2203), while rRNAs and tRNAs generally showed positive values (Figure 3). GC‐skew values were consistently negative (−0.1683 to −0.1487) across complete mitogenomes, whereas rRNAs and tRNAs exhibited positive GC‐skews (0.11–0.28).

### 3.3 Protein‐Coding Genes and Codon Usage

All six mitogenomes contained the typical set of 13 PCGs. Nine genes (*ND2*, *COX1–3*, *ATP6*, *ATP8*, *CYTB*, *ND3*, and *ND6*) were located on the majority (J) strand, while four (*ND1*, *ND4*, *ND4L*, and *ND5*) were encoded on the minority (N) strand, consistent with other mayfly species (Figure 1). The total length of the 13 PCGs ranged from 11,200 bp in *T. jinghongensis* to 11,228 bp in *C. gosei*, accounting for approximately 71.68%–72.83% of the total mitogenome length.

Most PCGs started with conventional start codons (ATN: ATG, ATT, or ATA), typical for insects (Tables S1–, S6). Several non‐canonical start codons were also identified. *COX1* consistently used CGA as a start codon in all six species, as reported previously (Xu et al. 2020; Li et al. 2020). *ND6* initiated with TTG in *D. ishiyamana*, *T. nepalica*, *U. punctisetae*, and *C. femorata* , while *ND3* and *ND5* began with GTG in *U. punctisetae* and *T. jinghongensis*. For termination codons, most PCGs ended with TAA or TAG, though incomplete stop codons consisting of a single thymine (T) were found in several genes (*COX2*, *CYTB*, *ND4*, *ND5*), consistent with the pattern observed in many insect mitogenomes (Ojala et al. 1981).

**FIGURE 4:**
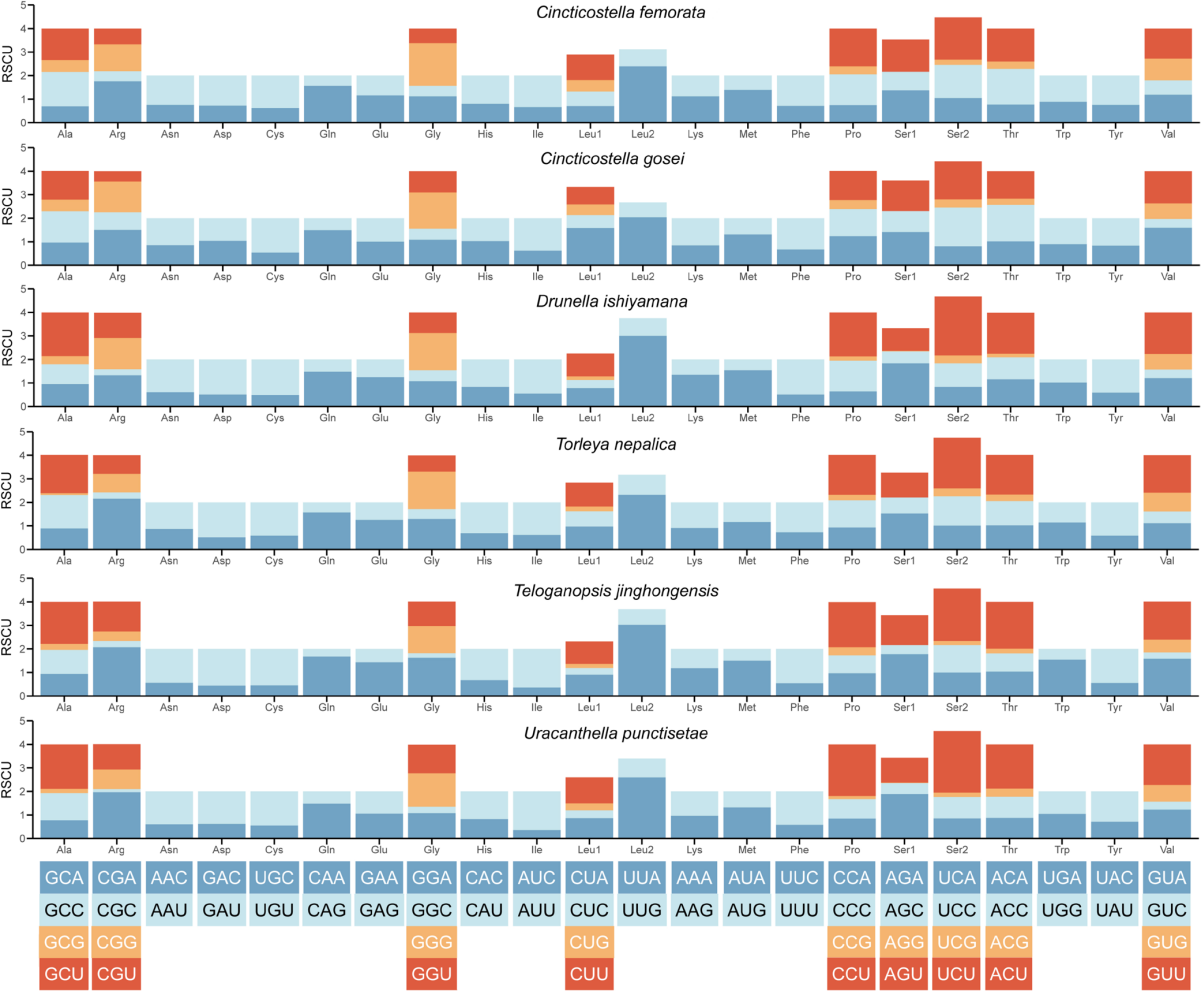
Relative synonymous codon usage (RSCU) of 12 Ephemerellidae mitogenomes. Codon families are provided on the *x*‐axis along with the different combinations of synonymous codons that code for that amino acid. RSCU is defined on the *y*‐axis.

Excluding stop codons, the total number of amino acids encoded by the 13 PCGs ranged from 3721 in *T. nepalica* to 3731 in *C. gosei*. The AGG codon was absent in four species and appeared only once in *U. punctisetae* and *D. ishiyamana*, indicating strong codon usage bias. Relative synonymous codon usage (RSCU) analysis revealed that the most frequently used codons were UUA (Leu), UUU (Phe), AUU (Ile), and AUA (Met), with a clear preference for codons ending in A or U (Figure 4).

### 3.4 Ribosomal and Transfer RNAs

All six mitogenomes contained two rRNA genes (rrnL and rrnS), both encoded on the N‐strand. *rrnL* was located between *trnL1* (CUN) and *trnV*, and *rrnS* between *trnV* and *trnI*, as observed in other Ephemerellidae species (Figure 1; Tables S1–, S6). The length of *rrnL* ranged from 1215 bp (*T. nepalica*) to 1230 bp (*T. jinghongensis*), while *rrnS* ranged from 777 bp (*T. nepalica*) to 838 bp (*U. punctisetae*). Both rRNAs were strongly A + T‐biased (63.70%–68.59%).

A total of 22 tRNA genes were identified in all mitogenomes, ranging from 61 to 72 bp in length. Secondary structure predictions showed that 21 tRNAs folded into the canonical cloverleaf structure, whereas *trnS1* (AGN) lacked a DHU arm and formed a simple loop, as commonly observed in insects including Heptageniidae and Baetidae (Figures S1–S6) (Cameron 2014). Several non‐standard base pairs (G–U, U–U, C–U, and C–A) were observed in tRNA stems (Varani and McClain 2000).

### 3.5 Non‐Coding Regions

Variation in the number and size of non‐coding regions was observed in all six Ephemerellidae mitogenomes. In each species, the control region (CR) was located between *rrnS* and *trnI*. CR lengths varied substantially, from 317 bp in *T. nepalica* to 954 bp in *T. jinghongensis*, representing the main source of interspecific size variation (Tables S1–, S6).

In addition to the CR, 6–10 intergenic spacer (IGS) regions were identified, ranging from 1 to 1212 bp in length. *D. ishiyamana* contained the longest IGS (1212 bp) between *ND4L* and *trnT*, largely explaining its extended mitogenome length (Tables S1–, S6). Gene overlaps ranged from 1 to 16 bp, with 13 junctions in *T. nepalica* and 16 in *C. gosei*. The overlapping pairs *ATP8*–*ATP6* (4 bp) and *ND4*–*ND4L* (7 bp) were conserved in all species, whereas *C. gosei* exhibited the largest overlap (16 bp) between *trnP* and *ND6*.

### 3.6 Comparative Analysis of Ephemerellidae Mitogenomes

Across the twelve available Ephemerellidae mitogenomes, including the six newly sequenced species, a strong A + T bias (60.32%–66.53%) was consistently observed (Table S7). Among genomic regions, PCGs had lower A + T contents (59.35%–66.24%) compared with tRNAs (61.08%–69.26%) and rRNAs (63.11%–68.70%) (Figure 2). The third codon positions consistently displayed higher A + T contents than the first and second positions.

**FIGURE 5:**
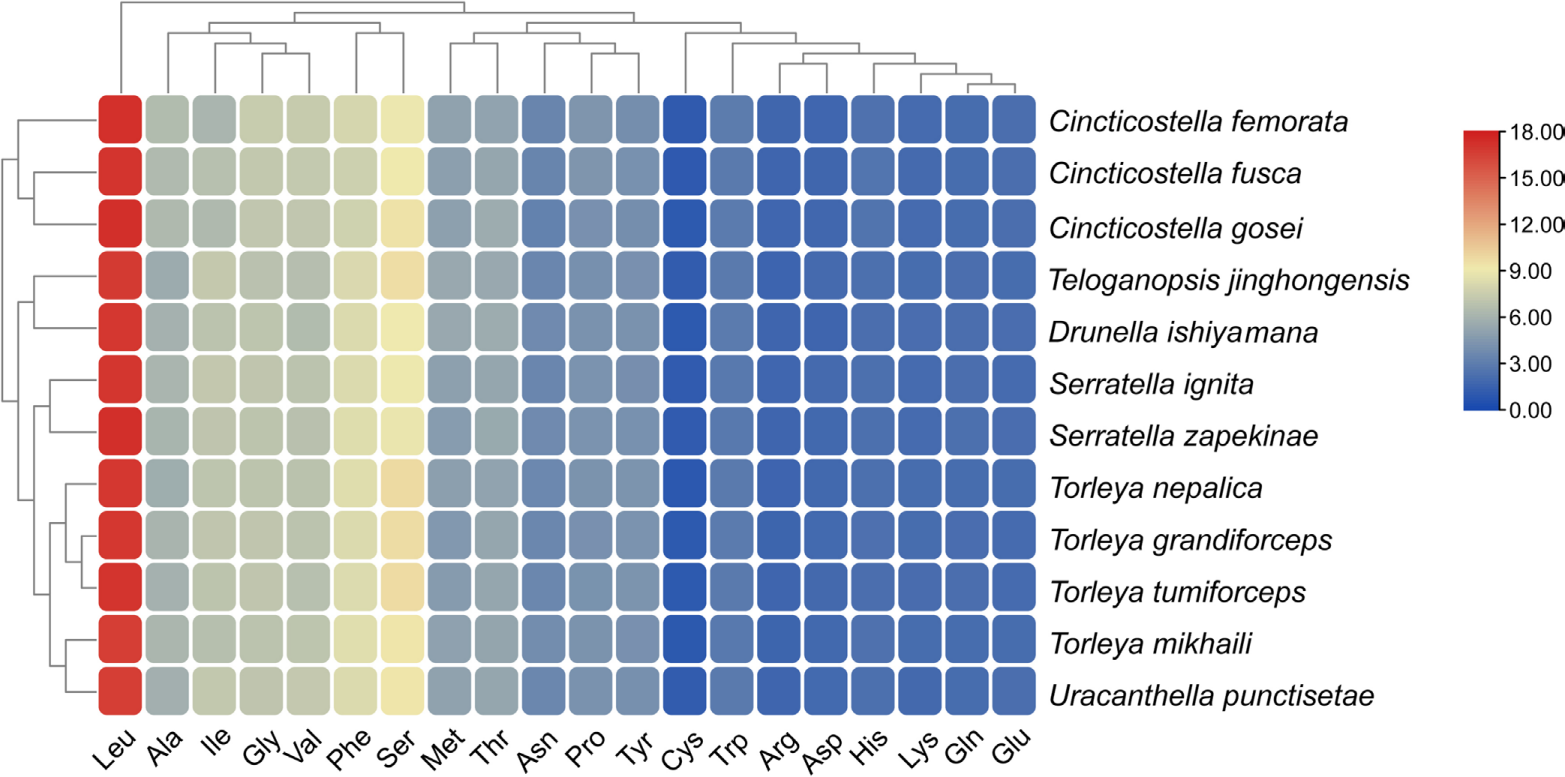
Amino acid composition of all the PCGs of 12 Ephemerellidae mitogenomes.

AT‐ and GC‐skew analyses revealed consistent compositional asymmetry across species (Figure 3). AT‐skews were generally negative for whole mitogenomes and PCGs but positive for tRNAs and rRNAs, whereas GC‐skews were predominantly negative except in RNA regions. Codon usage patterns were highly conserved among species, with a strong bias toward A/T‐ending codons, particularly in frequently used amino acids such as leucine (UUA/UUG), phenylalanine (UUU/UUC), and isoleucine (AUU/AUC) (Figure 5).

**FIGURE 6:**
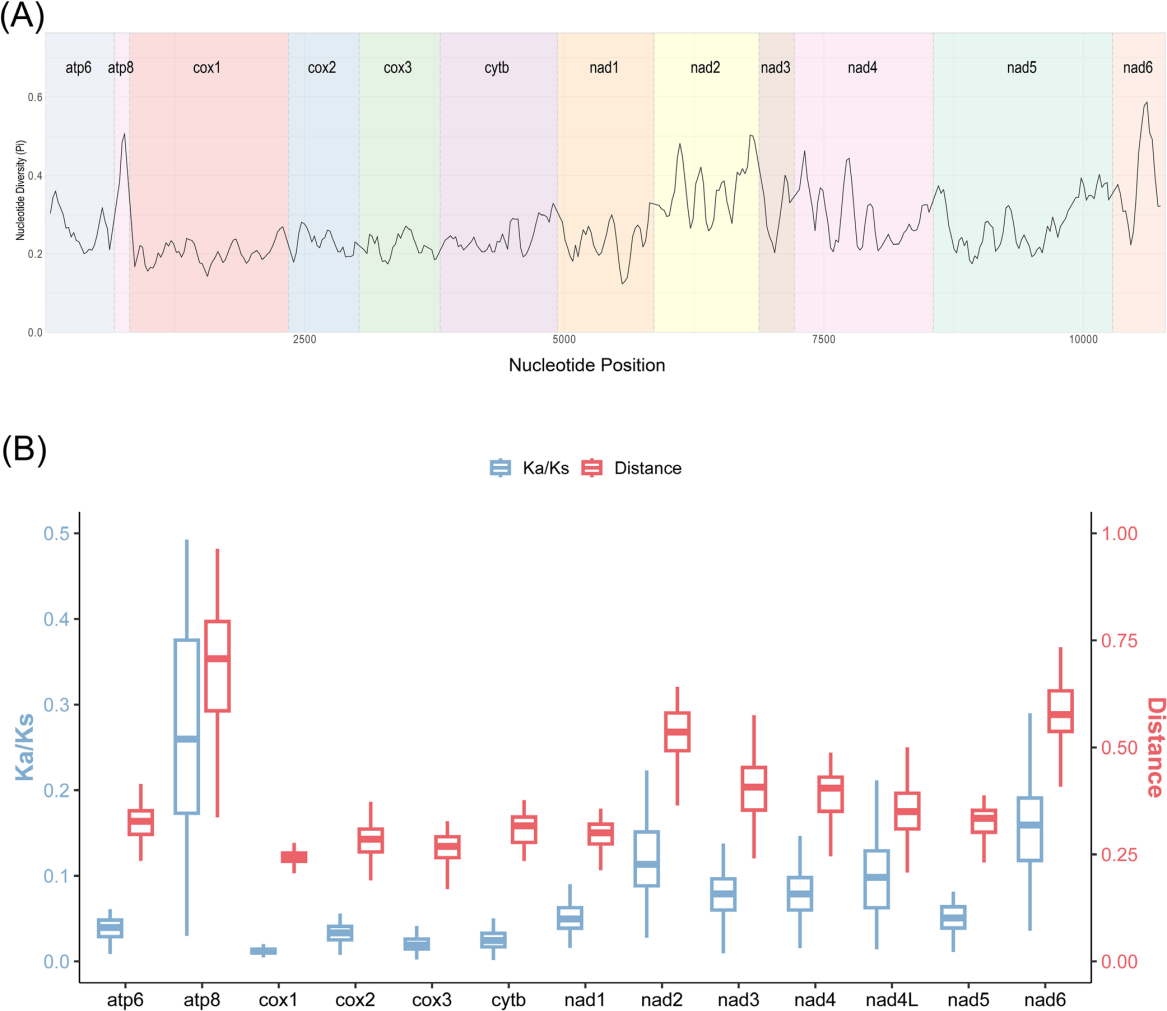
(A) Sliding window analysis based on 13 aligned PCGs. The line shows the value of the nucleotide diversity Pi. (B) Non‐synonymous (Ka) to synonymous (Ks) substitution rates of 13 PCGs among 12 Ephemerellidae species.

Nucleotide diversity (Pi) analyses revealed substantial heterogeneity among genes (Figure 6A). *ATP8* (Pi = 0.4578), *ND6* (0.389), and *ND2* (0.3641) exhibited the highest variability, whereas *COX1* (0.2033) and *COX2* (0.2287) were the most conserved. Genetic distance analyses yielded consistent results (Figure 6B), with *ATP8* (0.7615), *ND6* (0.5732), and *ND2* (0.5246) showing the highest substitution rates. Ka/Ks ratios ranged from 0.0132 (*COX1*) to 0.3264 (*ATP8*), all below 1, suggesting pervasive purifying selection across all PCGs.

### 3.7 Phylogenetic Analysis

**FIGURE 7:**
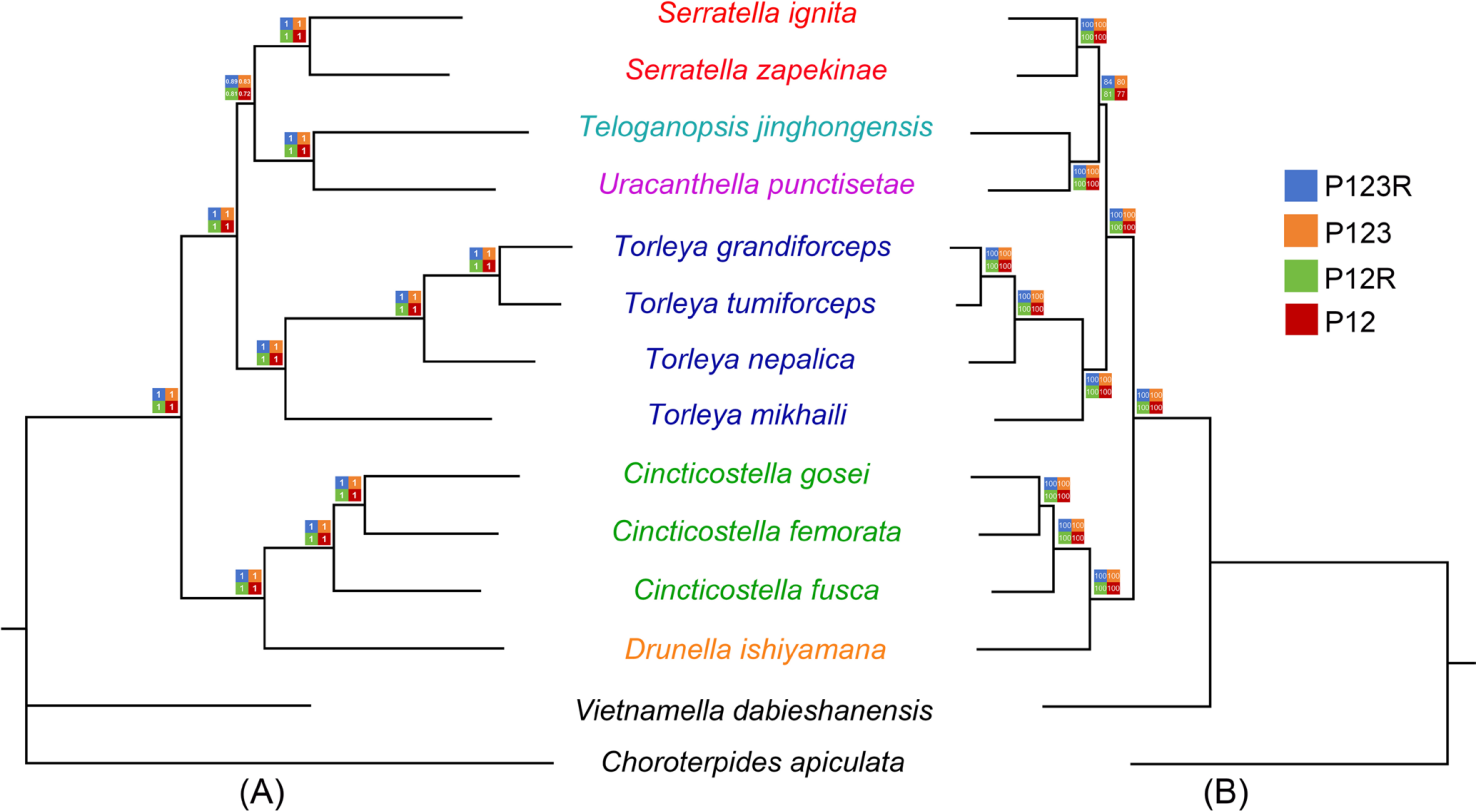
Phylogenetic analyses of Ephemerellidae with Bayesian inference (A) and Maximum likelihood (B). Numbers separated by a slash on the nodes represent the posterior probability (PP) and bootstrap value (BV) of different datasets.

Phylogenetic analyses based on the concatenated sequences of 13 PCGs and two rRNAs from 12 Ephemerellidae species, together with two outgroup taxa from Leptophlebiidae and Vietnamellidae, were conducted using Bayesian inference (BI) and maximum likelihood (ML) methods (Figure 7). Four datasets were analyzed, namely P123R (13 PCGs + 2 rRNAs, 11,631 bp), P123 (13 PCGs, 10,170 bp), P12R (first and second codon positions + 2 rRNAs, 8422 bp), and P12 (first and second codon positions only, 6810 bp).

Consistent with the traditional classification proposed by McCafferty (2000), all six genera of Ephemerellidae included in this study (*Drunella*, *Teloganopsis*, *Torleya*, *Uracanthella*, *Cincticostella*, and *Serratella*) are assigned to the subfamily Ephemerellinae. The phylogenetic trees generated from all datasets using both analytical methods exhibited congruent topologies and recovered three well‐defined clades within Ephemerellinae (Figure 7). The three genera represented by more than two species were each resolved as monophyletic with high posterior probabilities and bootstrap values. In both analyses, *T. jinghongensis* and *U. punctisetae* clustered together with strong support and formed a sister group to *Serratella*.

## 4 Discussion

The six newly sequenced mitogenomes expanded the mitochondrial genomic resources available for Ephemerellidae and revealed several characteristic features of genome organization and evolution in this family. The observed gene content and overall genome size fall within the range typical of mayflies (Li et al. 2014; Gao et al. 2018), but pronounced variation in total length, largely driven by differences in control region and intergenic spacer lengths, highlights the dynamic nature of non‐coding regions in Ephemerellidae mitogenomes. A striking feature of the new sequences is the *trnI*‐associated rearrangement. All six mitogenomes share an inverted and translocated *trnI* that has shifted from the ancestral CR–*trnQ*–*trnM* region to a position between *rrnS* and the CR, and *T. nepalica* contains four copies of *trnI*. Together with previous reports of *trnI* inversion, duplication, and multiple copies in *Ephemerella*, *Serratella*, and *Torleya* (Xu et al. 2020; Li et al. 2020), these data strongly support *trnI*‐associated rearrangements as a synapomorphic feature of Ephemerellidae mitogenomes. The combination of duplication and positional changes is consistent with the tandem duplication–random loss (TDRL) model that has been widely invoked to explain mitochondrial gene rearrangements in insects (Xu et al. 2020; Li et al. 2020; Clary and Wolstenholme 1985). The presence of lineage‐specific duplications in *Torleya* suggests that TDRL‐like processes may have occurred repeatedly during the diversification of the family.

All newly sequenced mitogenomes exhibit a strong A + T bias, with especially high A + T contents in rRNAs and at third codon positions (Figure 2). Similar patterns have been documented in other Ephemeroptera and many insect orders (Xu et al. 2020; Li et al. 2020; Li et al. 2025). The consistent negative AT‐ and GC‐skews in PCGs, contrasted with positive skews in RNA genes, indicate stable yet region‐specific compositional asymmetry. Such asymmetry is typically attributed to strand‐specific mutational biases associated with asymmetric replication of the mitochondrial genome and may influence both codon usage and substitution patterns over evolutionary time. Codon usage analyses revealed a strong preference for A/T‐ending codons and an extremely low frequency of some GC‐rich codons (e.g., AGG) (Figure 4). This pattern mirrors the overall A + T bias and is likely shaped by a combination of mutational bias and translational selection, which together favor codons that match the cellular tRNA pool and improve translational efficiency in the mitochondria. The pervasive use of codons such as UUA (Leu), UUU (Phe), AUU (Ile), and AUA (Met) suggests that codon usage in Ephemerellidae mitogenomes is highly constrained and conserved, providing a consistent background against which gene‐ and lineage‐specific changes can be detected (Shen et al. 2012).

The structure and evolution of non‐coding regions further illustrate the dynamic nature of ephemerellid mitogenomes. The broad range of CR lengths (317–954 bp) and the extensive intergenic spacer between *ND4L* and *trnT* in *D. ishiyamana* suggest frequent insertion–deletion events and tandem repeat expansion or contraction (Lv et al. 2018; Clayton 1991; Fernandez‐Silva et al. 2003). Such variation provides additional characters for comparative genomics and may contain regulatory motifs associated with replication and transcription. Future detailed analyses of repeat structure, secondary motifs, and substitution patterns in these regions may uncover lineage‐specific signatures linked to life‐history traits or environmental adaptation.

Comparative analyses of nucleotide diversity and evolutionary rates across the 13 PCGs revealed marked heterogeneity. *ATP8*, *ND6*, and *ND2* showed the highest levels of nucleotide diversity and genetic distance, whereas *COX1* and *COX2* were among the most conserved genes. Ka/Ks ratios for all PCGs were < 1, indicating that purifying selection is the dominant force acting on mitochondrial protein‐coding genes in Ephemerellidae. However, the relatively elevated ω values for *ATP8*, *ND6*, and *ND2* point to relaxed functional constraints or lineage‐specific adaptations in these genes. ATP8 is short, which can exaggerate apparent substitution rates, but *ND2* and *ND6* are of moderate length and display consistent patterns across analyses. Together with the widely used *COX1* barcode, *ND2* and *ND6* therefore represent promising supplementary markers for species delimitation, population genetic studies, and finer‐scale phylogenetic reconstruction within Ephemerellidae.

Phylogenetic analyses based on concatenated PCG and rRNA datasets recovered congruent and well‐supported topologies across BI and ML approaches and across different data partitions. The three genera represented by multiple species (*Torleya*, *Cincticostella*, and *Serratella*) were each resolved as monophyletic, corroborating their current generic definitions and supporting previous mitogenomic and multi‐locus studies (Xu et al. 2020; Li et al. 2020; McCafferty 2000). The consistent placement of *T. jinghongensis* and *U. punctisetae* as sister taxa, with this clade in turn sister to *Serratella*, agrees with morphological hypotheses and earlier molecular results (Ogden et al. 2009). Nonetheless, the moderate support for some deeper nodes is likely attributable to limited taxon sampling, as exemplified by the inclusion of only a single species for genera such as *Teloganopsis* and *Uracanthella*, coupled with the overall scarcity of complete mitogenomes currently available across relevant genera and species.

Overall, the mitogenomic evidence presented here provides new insights into mitogenome evolution and phylogenetic relationships within Ephemerellidae. The characteristic *trnI* rearrangements, strong and region‐specific compositional biases, heterogeneous evolutionary rates across PCGs, and robustly resolved generic relationships collectively highlight the value of complete mitogenomes for studying this ecologically and taxonomically important mayfly family. However, Ephemerellidae remains underrepresented in mitochondrial datasets compared with other mayfly families such as Heptageniidae. Expanding taxon sampling to include additional genera, species, and geographically distinct populations—ideally combining mitogenomic data with nuclear markers—will be essential for constructing a more comprehensive and stable phylogenetic framework and for testing alternative hypotheses regarding the origin and diversification of Ephemerellidae.

## Author Contributions

**Chao Xue:** data curation (equal), formal analysis (equal), software (equal), validation (equal), writing – original draft (lead), writing – review and editing (lead). **Zhenxing Ma:** resources (equal), software (equal), validation (equal). **Dongkai Liu:** formal analysis (equal), investigation (equal), methodology (equal), validation (equal). **Ran Li:** conceptualization (equal), funding acquisition (lead), methodology (lead), project administration (equal), supervision (equal), visualization (equal), writing – original draft (equal), writing – review and editing (equal). **Xianfeng Yi:** investigation (equal), methodology (equal), project administration (equal), writing – original draft (equal), writing – review and editing (equal).

## Funding

This work was supported by the National Natural Science Foundation of China, grant number 32200359; and the China Postdoctoral Science Foundation, grant number 2024M751749.

## Conflicts of Interest

The authors declare no conflicts of interest.

## Supporting information


**Figure S1:** The putative tRNA secondary structure of *Teloganopsis jinghongensis*.
**Figure S2:** The putative tRNA secondary structure of *Torleya nepalica*.
**Figure S3:** The putative tRNA secondary structure of *Drunella ishiyamana*.
**Figure S4:** The putative tRNA secondary structure of *Cincticostella gosei*.
**Figure S5:** The putative tRNA secondary structure of *Uracanthella punctisetae*.
**Figure S6:** The putative tRNA secondary structure of *Cincticostella femorata*.

**Table S1:** Annotation and gene organization of the *Cincticostella femorata* mitogenome.

**Table S2:** Annotation and gene organization of the *Cincticostella gosei* mitogenome.

**Table S3:** Annotation and gene organization of the *Drunella ishiyamana* mitogenome.

**Table S4:** Annotation and gene organization of the *Teloganopsis jinghongensis* mitogenome.

**Table S5:** Annotation and gene organization of the *Torleya nepalica* mitogenome.

**Table S6:** Annotation and gene organization of the *Uracanthella punctisetae* mitogenome.

**Table S7:** A + T content (%) of different mitochondrial genomic regions in Ephemerellidae species.

**Table S8:** AT‐ and GC‐skew values of different mitochondrial genomic regions in six newly sequenced Ephemerellidae species.

## Data Availability

The mitogenomes have been deposited in the GenBank database under the accession numbers: PX508687 (*Teloganopsis jinghongensis*), PX508688 (*Torleya nepalica*), PX508690 (*Drunella ishiyamana*), PX508691 (*Cincticostella gosei*), PX508692 (*Uracanthella punctisetae*) and PX508693 (*Cincticostella femorata*).
