# Supplementary material for: Mitochondrial Genome Characterization of Six Spiny Crawler Mayflies and Comparative Analysis Within Ephemerellidae (Ephemeroptera: Pannota)
Source: Ecol Evol. 2026 Jan 8;16(1):e72935. doi: 10.1002/ece3.72935 (PMC12782776; doi:10.1002/ece3.72935)
Supplement: Supplementary file 1 — Figure S1: The putative tRNA secondary structure of Teloganopsis jinghongensis. Figure S2: The putative tRNA secondary structure of Torleya nepalica. Figure S3: The putative tRNA secondary structure of Drunella ishiyamana. Figure S4: The putative tRNA secondary structure of Cincticostella gosei. Figure S5: The putative tRNA secondary structure of Uracanthella punctisetae. Figure S6: The putative tRNA secondary structure of Cincticostella femorata. [file ECE3-16-e72935-s008.docx]

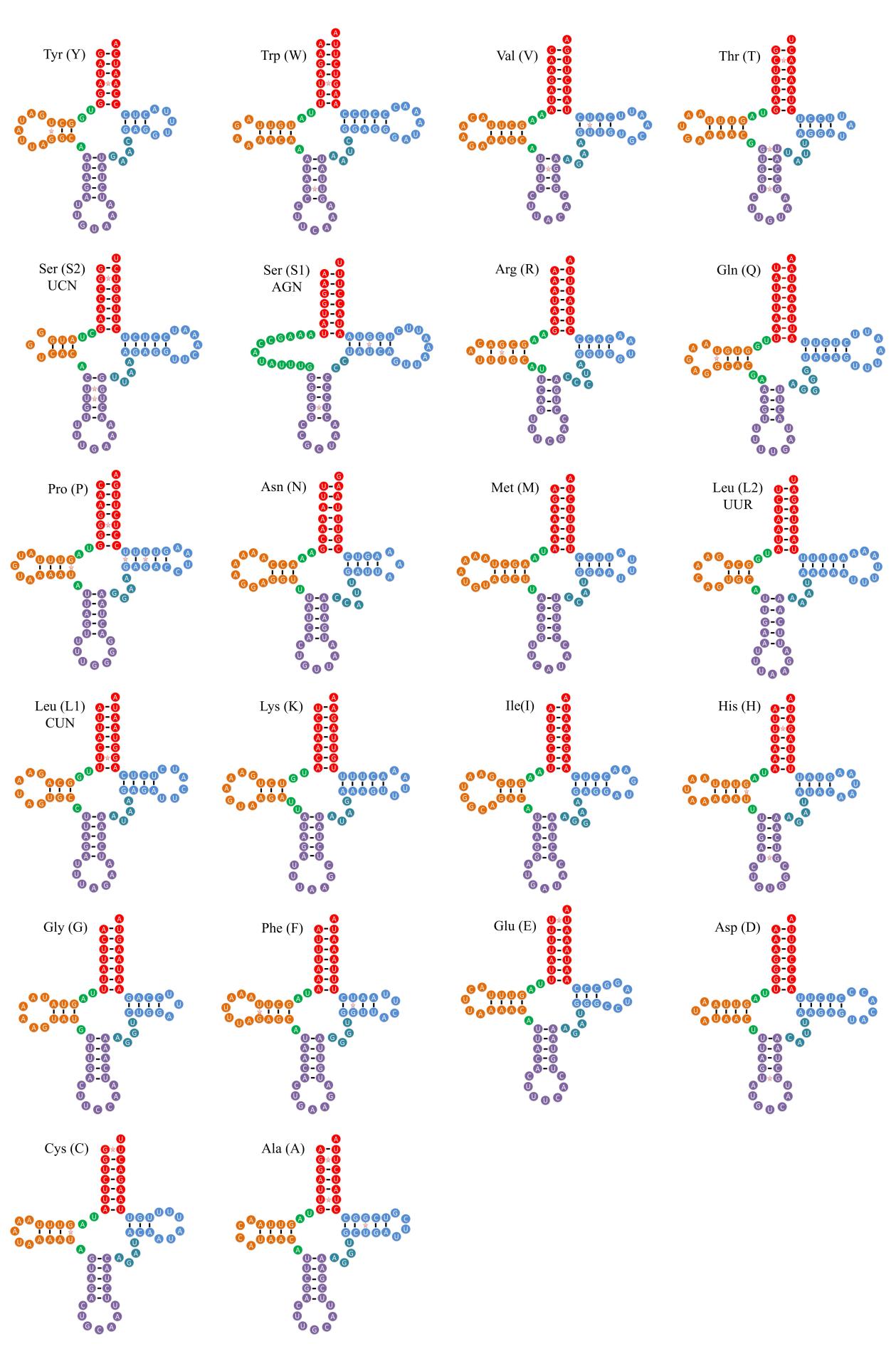


**Figure S1.** The putative tRNA secondary structure of *Teloganopsis jinghongensis.*


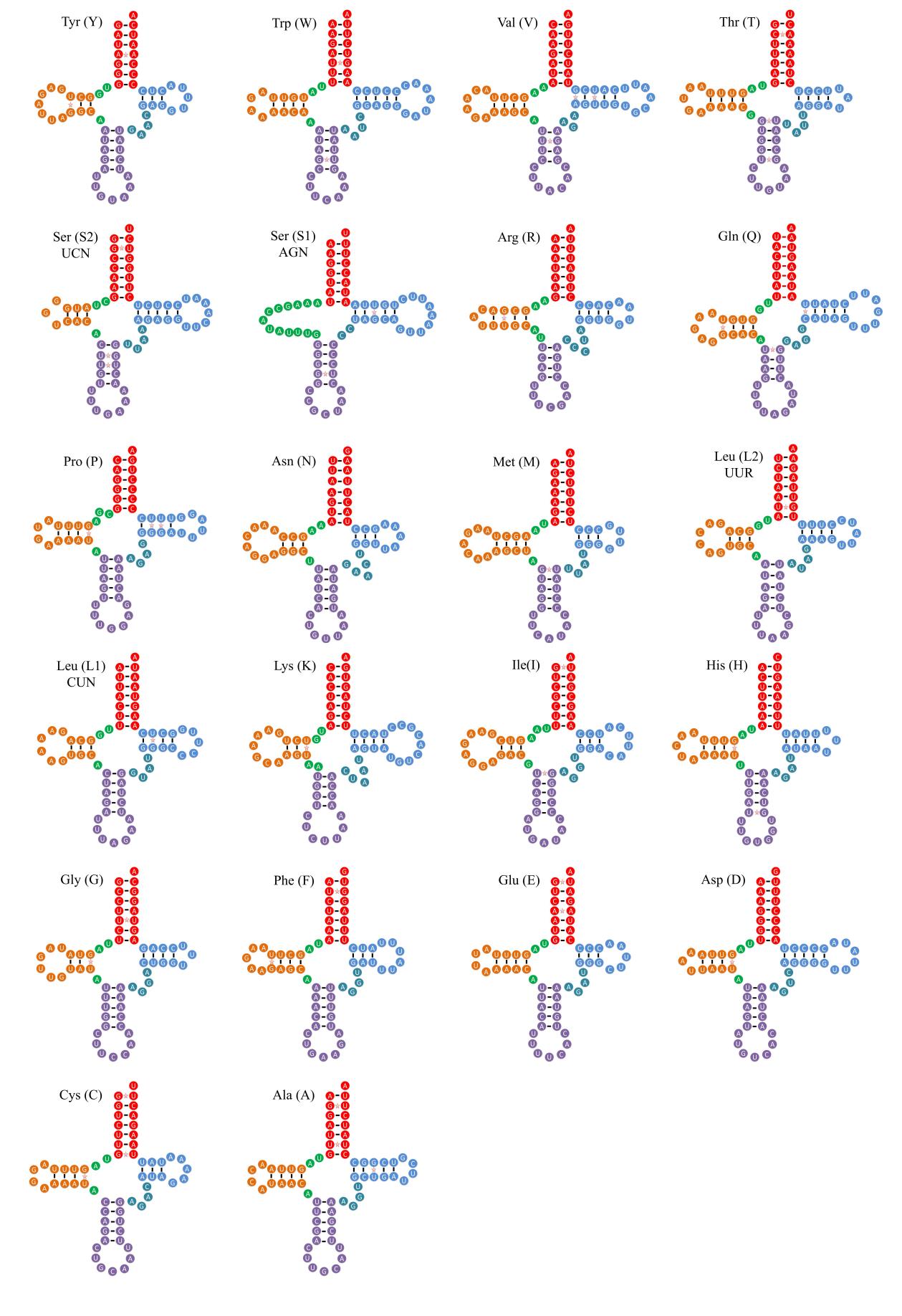


**Figure S2.** The putative tRNA secondary structure of *Torleya nepalica.*


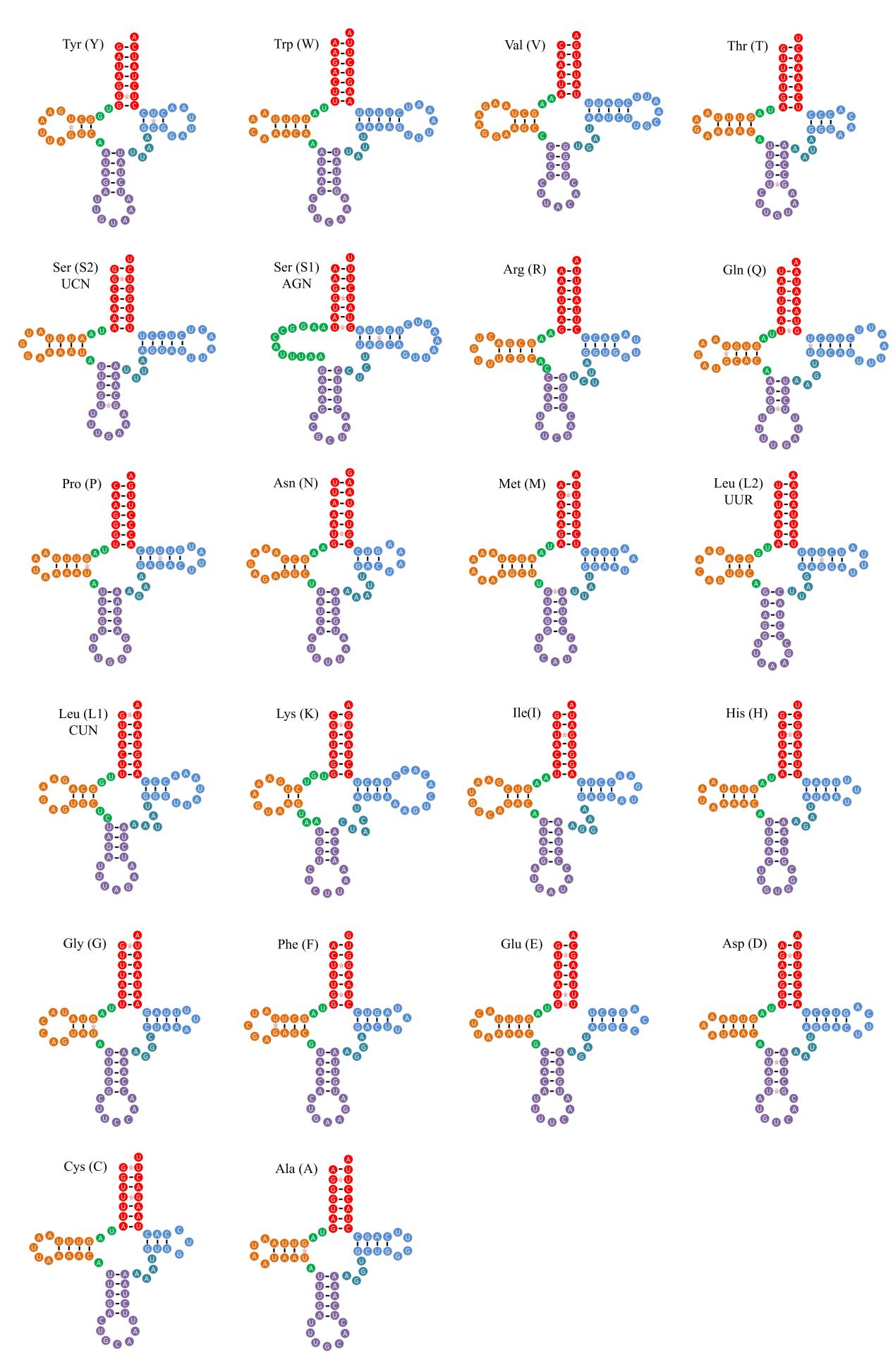


**Figure S3.** The putative tRNA secondary structure of *Drunella ishiyamana.*


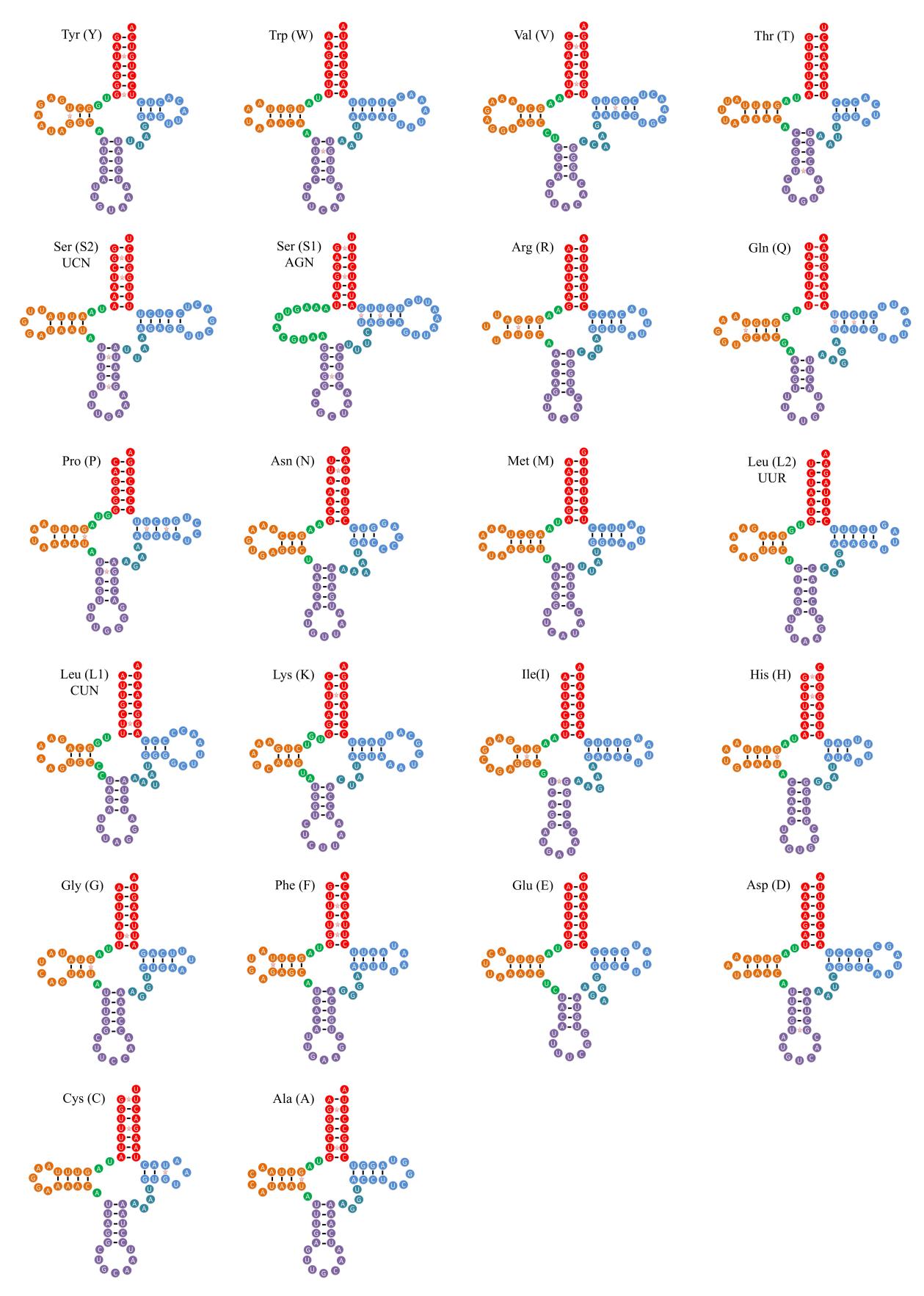


**Figure S4.** The putative tRNA secondary structure of *Cincticostella gosei.*


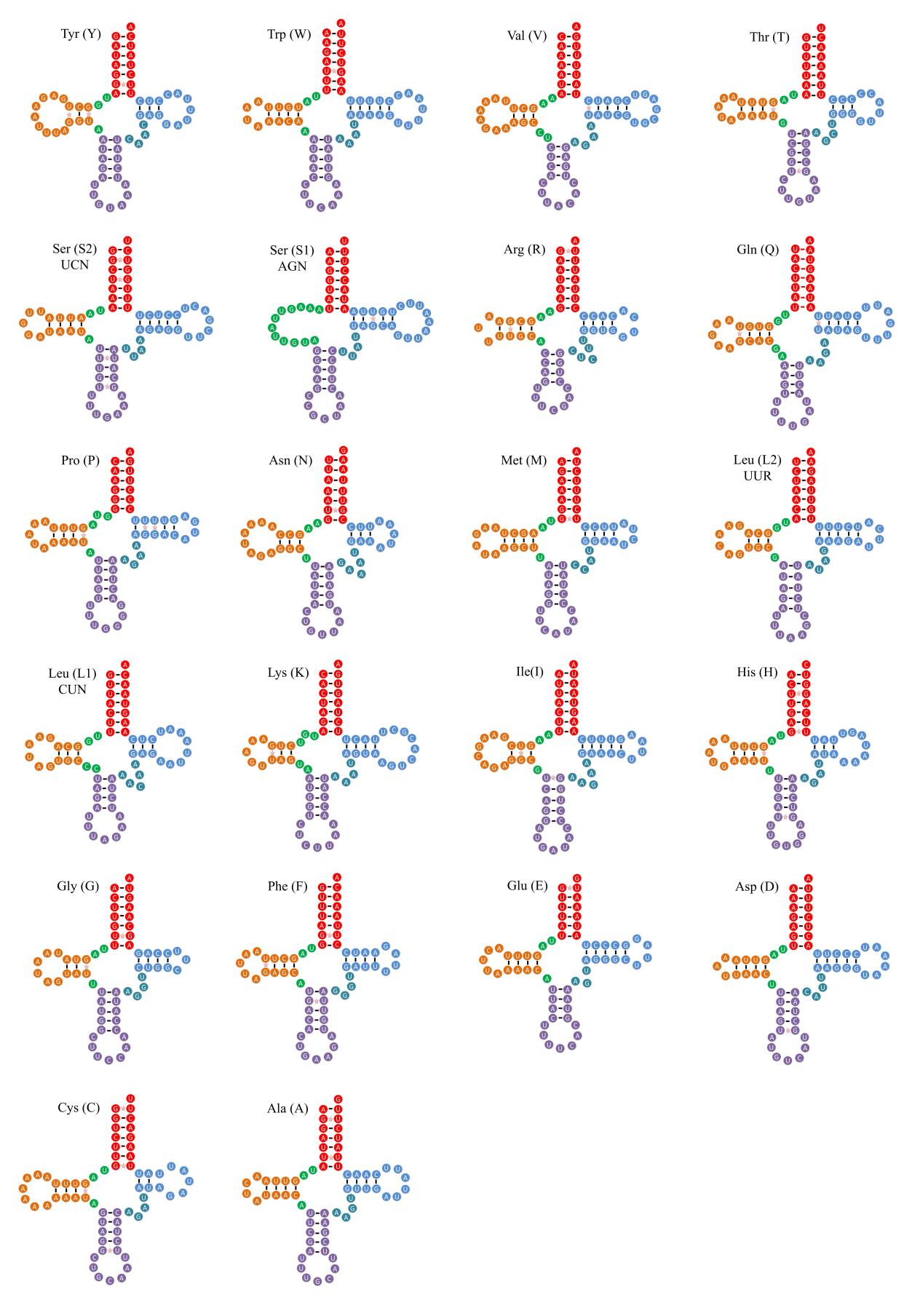


**Figure S5.** The putative tRNA secondary structure of *Uracanthella punctisetae.*


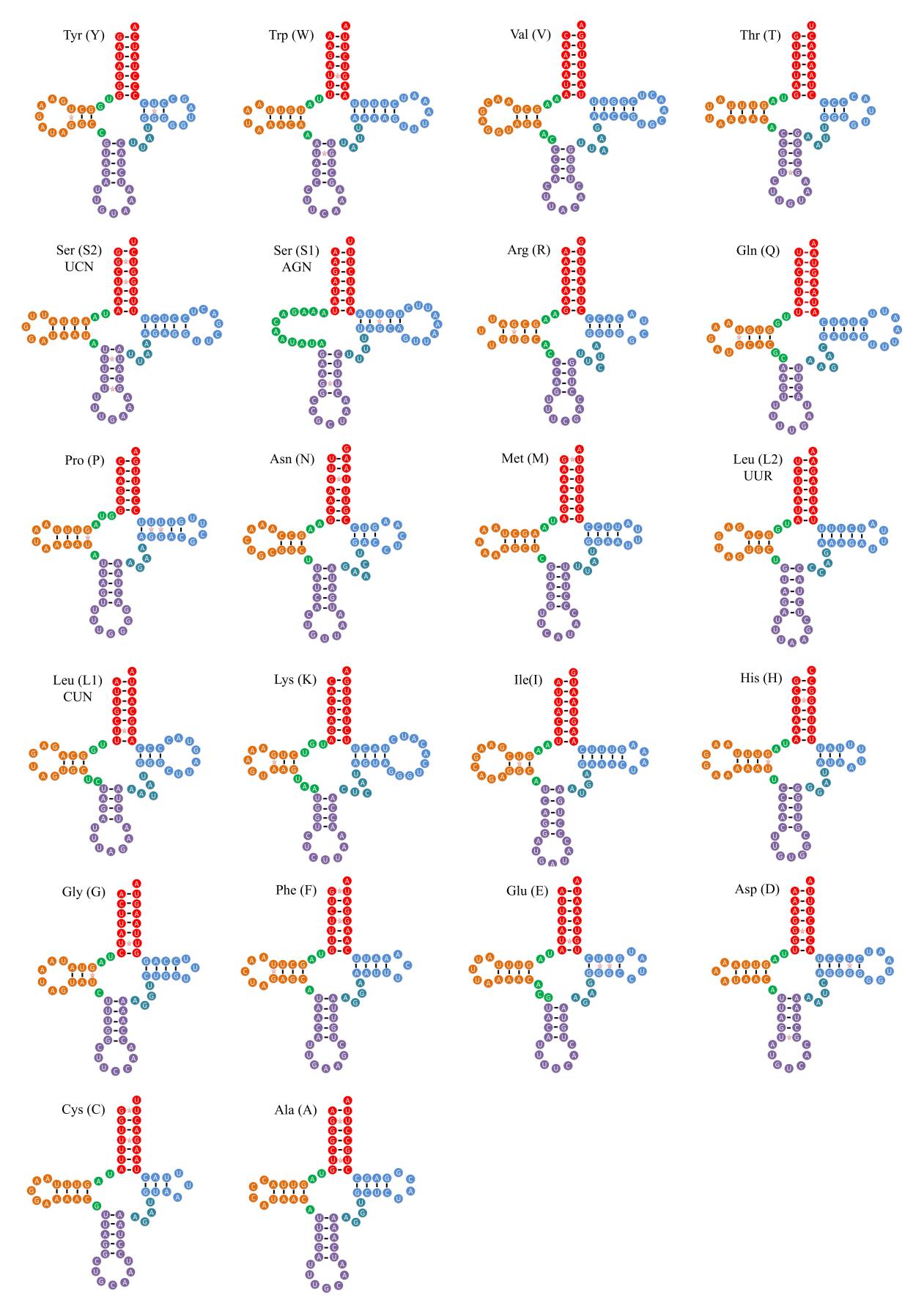


**Figure S6.** The putative tRNA secondary structure of *Cincticostella femorata.*
