# Supplementary material for: Mitochondrial Genome Characterization of Six Spiny Crawler Mayflies and Comparative Analysis Within Ephemerellidae (Ephemeroptera: Pannota)
Source: Ecol Evol. 2026 Jan 8;16(1):e72935. doi: 10.1002/ece3.72935 (PMC12782776; doi:10.1002/ece3.72935)
Supplement: Supplementary file 2 — Table S1: Annotation and gene organization of the Cincticostella femorata mitogenome. [file ECE3-16-e72935-s003.docx]

**Table S1.** Annotation and gene organization of the *Cincticostella femorata* mitogenome.

| **Gene** | **Strand** | **Nucleotide no.** | **Size(bp)** | **IN** | **Anticodon** | **Start codon** | **Stop codon** |
| --- | --- | --- | --- | --- | --- | --- | --- |
| *trnI* | N | 1-66 | 66 | 0 | GAT |  |  |
| AT-rich | J | 67-808 | 742 | 0 |  |  |  |
| *trnQ* | N | 809-877 | 69 | 0 | TTG |  |  |
| *trnM* | J | 878-941 | 64 | 0 | CAT |  |  |
| *ND2* | J | 942-1964 | 1023 | 0 |  | ATT | TAA |
| *trnW* | J | 1963-2030 | 68 | -2 | TCA |  |  |
| *trnC* | N | 2023-2083 | 61 | -8 | GCA |  |  |
| *trnY* | N | 2084-2147 | 64 | 0 | GTA |  |  |
| *COX1* | J | 2149-3684 | 1536 | 1 |  | CGA | TAA |
| *trnL2* | J | 3680-3744 | 65 | -5 | TAA |  |  |
| *COX2* | J | 3746-4433 | 688 | 1 |  | ATG | T |
| *trnK* | J | 4434-4503 | 70 | 0 | CTT |  |  |
| *trnD* | J | 4503-4568 | 66 | -1 | GTC |  |  |
| *ATP8* | J | 4569-4733 | 165 | 0 |  | ATT | TAA |
| *ATP6* | J | 4730-5404 | 675 | -4 |  | ATA | TAA |
| *COX3* | J | 5404-6192 | 789 | -1 |  | ATG | TAA |
| *trnG* | J | 6195-6256 | 62 | 2 | TCC |  |  |
| *ND3* | J | 6257-6610 | 354 | 0 |  | TTG | TAG |
| *trnA* | J | 6609-6671 | 63 | -2 | TGC |  |  |
| *trnR* | J | 6671-6732 | 62 | -1 | TCG |  |  |
| *trnN* | J | 6730-6794 | 65 | -3 | GTT |  |  |
| *trnS1* | J | 6792-6858 | 67 | -3 | GCT |  |  |
| *trnE* | J | 6993-7056 | 64 | 134 | TTC |  |  |
| *trnF* | N | 7055-7117 | 63 | -2 | GAA |  |  |
| *ND5* | N | 7117-8856 | 1740 | -1 |  | ATA | TAG |
| *trnH* | N | 8857-8919 | 63 | 0 | GTG |  |  |
| *ND4* | N | 8919-10,265 | 1347 | -1 |  | ATG | TAG |
| *ND4L* | N | 10,259-10,555 | 297 | -7 |  | ATG | TAA |
| *trnT* | J | 10,612-10,674 | 63 | 56 | TGT |  |  |
| *trnP* | N | 10,675-10,739 | 65 | 0 | TGG |  |  |
| *ND6* | J | 10,742-11,260 | 519 | 2 |  | TTG | TAA |
| *CYTB* | J | 11,260-12,394 | 1135 | -1 |  | ATG | T |
| *trnS2* | J | 12,395-12,463 | 69 | 0 | TGA |  |  |
| *ND1* | N | 12,481-13,419 | 939 | 17 |  | ATG | TAA |
| *trnL1* | N | 13,421-13,485 | 65 | 1 | TAG |  |  |
| *rrnL* | N | 13,486-14,711 | 1226 | 0 |  |  |  |
| *trnV* | N | 14,712-14,781 | 70 | 0 | TAC |  |  |
| *rrnS* | N | 14,782-15,594 | 813 | 0 |  |  |  |

Note: IN: Length of intergenic spacer, negative values indicate gene overlap.
