# Supplementary material for: Mitochondrial Genome Characterization of Six Spiny Crawler Mayflies and Comparative Analysis Within Ephemerellidae (Ephemeroptera: Pannota)
Source: Ecol Evol. 2026 Jan 8;16(1):e72935. doi: 10.1002/ece3.72935 (PMC12782776; doi:10.1002/ece3.72935)
Supplement: Supplementary file 3 — Table S2: Annotation and gene organization of the Cincticostella gosei mitogenome. [file ECE3-16-e72935-s004.docx]

**Table S2.** Annotation and gene organization of the *Cincticostella gosei* mitogenome.

| **Gene** | **Strand** | **Nucleotide no.** | **Size(bp)** | **IN** | **Anticodon** | **Start codon** | **Stop codon** |
| --- | --- | --- | --- | --- | --- | --- | --- |
| *trnI* | N | 1-65 | 65 | 0 | GAT |  |  |
| AT-rich | J | 66-760 | 695 | 0 |  |  |  |
| *trnQ* | N | 761-829 | 69 | 0 | TTG |  |  |
| *trnM* | J | 830-893 | 64 | 0 | CAT |  |  |
| *ND2* | J | 894-1916 | 1023 | 0 |  | ATT | TAA |
| *trnW* | J | 1915-1982 | 68 | -2 | TCA |  |  |
| *trnC* | N | 1975-2035 | 61 | -8 | GCA |  |  |
| *trnY* | N | 2036-2098 | 63 | 0 | GTA |  |  |
| *COX1* | J | 2100-3635 | 1536 | 1 |  | CGA | TAA |
| *trnL2* | J | 3631-3695 | 65 | -5 | TAA |  |  |
| *COX2* | J | 3697-4384 | 688 | 1 |  | ATG | T |
| *trnK* | J | 4385-4452 | 68 | 0 | CTT |  |  |
| *trnD* | J | 4452-4517 | 66 | -1 | GTC |  |  |
| *ATP8* | J | 4518-4682 | 165 | 0 |  | ATC | TAA |
| *ATP6* | J | 4679-5353 | 675 | -4 |  | ATA | TAA |
| *COX3* | J | 5353-6141 | 789 | -1 |  | ATG | TAA |
| *trnG* | J | 6144-6205 | 62 | 2 | TCC |  |  |
| *ND3* | J | 6206-6559 | 354 | 0 |  | TTG | TAG |
| *trnA* | J | 6558-6621 | 64 | -2 | TGC |  |  |
| *trnR* | J | 6621-6683 | 63 | -1 | TCG |  |  |
| *trnN* | J | 6681-6745 | 65 | -3 | GTT |  |  |
| *trnS1* | J | 6743-6809 | 67 | -3 | GCT |  |  |
| *trnE* | J | 6810-6873 | 64 | 0 | TTC |  |  |
| *trnF* | N | 6872-6934 | 63 | -2 | GAA |  |  |
| *ND5* | N | 6934-8673 | 1740 | -1 |  | ATA | TAG |
| *trnH* | N | 8674-8736 | 63 | 0 | GTG |  |  |
| *ND4* | N | 8736-10,082 | 1347 | -1 |  | ATG | TAG |
| *ND4L* | N | 10,076-10,372 | 297 | -7 |  | ATG | TAA |
| *trnT* | J | 10,435-10,496 | 62 | 62 | TGT |  |  |
| *trnP* | N | 10,497-10,561 | 65 | 0 | TGG |  |  |
| *ND6* | J | 10,546-11,085 | 540 | -16 |  | ATT | TAA |
| *CYTB* | J | 11,085-12,219 | 1135 | -1 |  | ATG | T |
| *trnS2* | J | 12,220-12,288 | 69 | 0 | TGA |  |  |
| *ND1* | N | 12,306-13,244 | 939 | 17 |  | ATG | TAA |
| *trnL1* | N | 13,246-13,310 | 65 | 1 | TAG |  |  |
| *rrnL* | N | 13,311-14,535 | 1225 | 0 |  |  |  |
| *trnV* | N | 14,536-14,605 | 70 | 0 | TAC |  |  |
| *rrnS* | N | 14,606-15,416 | 811 | 0 |  |  |  |

Note: IN: Length of intergenic spacer, negative values indicate gene overlap.
