# Supplementary material for: Mitochondrial Genome Characterization of Six Spiny Crawler Mayflies and Comparative Analysis Within Ephemerellidae (Ephemeroptera: Pannota)
Source: Ecol Evol. 2026 Jan 8;16(1):e72935. doi: 10.1002/ece3.72935 (PMC12782776; doi:10.1002/ece3.72935)
Supplement: Supplementary file 4 — Table S3: Annotation and gene organization of the Drunella ishiyamana mitogenome. [file ECE3-16-e72935-s005.docx]

**Table S3.** Annotation and gene organization of the *Drunella ishiyamana* mitogenome.

| **Gene** | **Strand** | **Nucleotide no.** | **Size(bp)** | **IN** | **Anticodon** | **Start codon** | **Stop codon** |
| --- | --- | --- | --- | --- | --- | --- | --- |
| *trnI* | N | 1-64 | 64 | 0 | GAT |  |  |
| AT-rich | J | 65-679 | 615 | 0 |  |  |  |
| *trnQ* | N | 680-748 | 69 | 0 | TTG |  |  |
| *trnM* | J | 750-814 | 65 | 1 | CAT |  |  |
| *ND2* | J | 815-1837 | 1023 | 0 |  | ATT | TAA |
| *trnW* | J | 1836-1903 | 68 | -2 | TCA |  |  |
| *trnC* | N | 1896-1956 | 61 | -8 | GCA |  |  |
| *trnY* | N | 1957-2019 | 63 | 0 | GTA |  |  |
| *COX1* | J | 2021-3556 | 1536 | 1 |  | CGA | TAA |
| *trnL2* | J | 3552-3616 | 65 | -5 | TAA |  |  |
| *COX2* | J | 3618-4305 | 688 | 1 |  | ATG | T |
| *trnK* | J | 4303-4372 | 70 | -3 | CTT |  |  |
| *trnD* | J | 4373-4436 | 64 | 0 | GTC |  |  |
| *ATP8* | J | 4437-4601 | 165 | 0 |  | ATC | TAA |
| *ATP6* | J | 4598-5272 | 675 | -4 |  | ATA | TAA |
| *COX3* | J | 5272-6060 | 789 | -1 |  | ATG | TAA |
| *trnG* | J | 6064-6125 | 62 | 3 | TCC |  |  |
| *ND3* | J | 6123-6479 | 357 | -3 |  | ATA | TAG |
| *trnA* | J | 6478-6540 | 63 | -2 | TGC |  |  |
| *trnR* | J | 6540-6603 | 64 | -1 | TCG |  |  |
| *trnN* | J | 6601-6663 | 63 | -3 | GTT |  |  |
| *trnS1* | J | 6661-6727 | 67 | -3 | GCT |  |  |
| *trnE* | J | 6728-6790 | 63 | 0 | TTC |  |  |
| *trnF* | N | 6789-6851 | 63 | -2 | GAA |  |  |
| *ND5* | N | 6853-8592 | 1740 | 1 |  | ATA | TAG |
| *trnH* | N | 8593-8654 | 62 | 0 | GTG |  |  |
| *ND4* | N | 8654-10,000 | 1347 | -1 |  | GTG | TAG |
| *ND4L* | N | 9994-10,290 | 297 | -7 |  | ATG | TAA |
| *trnT* | J | 11,503-11,563 | 61 | 1212 | TGT |  |  |
| *trnP* | N | 11,564-11,628 | 65 | 0 | TGG |  |  |
| *ND6* | J | 11,631-12,149 | 519 | 2 |  | TTG | TAA |
| *CYTB* | J | 12,149-13,285 | 1137 | -1 |  | ATG | TAA |
| *trnS2* | J | 13,287-13,354 | 68 | 1 | TGA |  |  |
| *ND1* | N | 13,371-14,309 | 939 | 16 |  | ATG | TAA |
| *trnL1* | N | 14,311-14,376 | 66 | 1 | TAG |  |  |
| *rrnL* | N | 14,377-15,598 | 1222 | 0 |  |  |  |
| *trnV* | N | 15,599-15,668 | 70 | 0 | TAC |  |  |
| *rrnS* | N | 15,669-16,485 | 817 | 0 |  |  |  |

Note: IN: Length of intergenic spacer, negative values indicate gene overlap.
