# Supplementary material for: Mitochondrial Genome Characterization of Six Spiny Crawler Mayflies and Comparative Analysis Within Ephemerellidae (Ephemeroptera: Pannota)
Source: Ecol Evol. 2026 Jan 8;16(1):e72935. doi: 10.1002/ece3.72935 (PMC12782776; doi:10.1002/ece3.72935)
Supplement: Supplementary file 5 — Table S4: Annotation and gene organization of the Teloganopsis jinghongensis mitogenome. [file ECE3-16-e72935-s001.docx]

**Table S4.** Annotation and gene organization of the *Teloganopsis jinghongensis* mitogenome.

| **Gene** | **Strand** | **Nucleotide no.** | **Size(bp)** | **IN** | **Anticodon** | **Start codon** | **Stop codon** |
| --- | --- | --- | --- | --- | --- | --- | --- |
| *trnI* | N | 1-64 | 64 | 0 | GAT |  |  |
| AT-rich | J | 65-1018 | 954 | 0 |  |  |  |
| *trnQ* | N | 1019-1087 | 69 | 0 | TTG |  |  |
| *trnM* | J | 1088-1154 | 67 | 0 | CAT |  |  |
| *ND2* | J | 1155-2177 | 1023 | 0 |  | ATT | TAA |
| *trnW* | J | 2176-2243 | 68 | -2 | TCA |  |  |
| *trnC* | N | 2236-2297 | 62 | -8 | GCA |  |  |
| *trnY* | N | 2298-2364 | 67 | 0 | GTA |  |  |
| *COX1* | J | 2366-3901 | 1536 | 1 |  | CGA | TAA |
| *trnL2* | J | 3897-3961 | 65 | -5 | TAA |  |  |
| *COX2* | J | 3963-4650 | 688 | 1 |  | ATG | T |
| *trnK* | J | 4651-4720 | 70 | 0 | CTT |  |  |
| *trnD* | J | 4720-4785 | 66 | -1 | GTC |  |  |
| *ATP8* | J | 4786-4947 | 162 | 0 |  | ATT | TAA |
| *ATP6* | J | 4944-5618 | 675 | -4 |  | ATA | TAA |
| *COX3* | J | 5618-6406 | 789 | -1 |  | ATG | TAA |
| *trnG* | J | 6406-6467 | 62 | -1 | TCC |  |  |
| *ND3* | J | 6468-6821 | 354 | 0 |  | GTG | TAG |
| *trnA* | J | 6820-6884 | 65 | -2 | TGC |  |  |
| *trnR* | J | 6884-6947 | 64 | -1 | TCG |  |  |
| *trnN* | J | 6945-7009 | 65 | -3 | GTT |  |  |
| *trnS1* | J | 7007-7073 | 67 | -3 | GCT |  |  |
| *trnE* | J | 7074-7137 | 64 | 0 | TTC |  |  |
| *trnF* | N | 7136-7201 | 66 | -2 | GAA |  |  |
| *ND5* | N | 7203-8942 | 1740 | 1 |  | GTG | TAA |
| *trnH* | N | 8943-9007 | 65 | 0 | GTG |  |  |
| *ND4* | N | 9007-10,353 | 1347 | -1 |  | ATG | TAA |
| *ND4L* | N | 10,347-10,643 | 297 | -7 |  | ATG | TAA |
| *trnT* | J | 10,649-10,711 | 63 | 5 | TGT |  |  |
| *trnP* | N | 10,712-10,777 | 66 | 0 | TGG |  |  |
| *ND6* | J | 10,783-11,298 | 516 | 5 |  | ATA | TAA |
| *CYTB* | J | 11,299-12,435 | 1137 | 0 |  | ATG | TAG |
| *trnS2* | J | 12,439-12,503 | 65 | 3 | TGA |  |  |
| *ND1* | N | 12,523-13,458 | 936 | 19 |  | ATG | TAA |
| *trnL1* | N | 13,462-13,526 | 65 | 3 | TAG |  |  |
| *rrnL* | N | 13,527-14,756 | 1230 | 0 |  |  |  |
| *trnV* | N | 14,757-14,825 | 69 | 0 | TAC |  |  |
| *rrnS* | N | 14,826-15,626 | 801 | 0 |  |  |  |

Note: IN: Length of intergenic spacer, negative values indicate gene overlap.
