# Supplementary material for: Mitochondrial Genome Characterization of Six Spiny Crawler Mayflies and Comparative Analysis Within Ephemerellidae (Ephemeroptera: Pannota)
Source: Ecol Evol. 2026 Jan 8;16(1):e72935. doi: 10.1002/ece3.72935 (PMC12782776; doi:10.1002/ece3.72935)
Supplement: Supplementary file 6 — Table S5: Annotation and gene organization of the Torleya nepalica mitogenome. [file ECE3-16-e72935-s002.docx]

**Table S5.** Annotation and gene organization of the *Torleya nepalica* mitogenome.

| **Gene** | **Strand** | **Nucleotide no.** | **Size(bp)** | **IN** | **Anticodon** | **Start codon** | **Stop codon** |
| --- | --- | --- | --- | --- | --- | --- | --- |
| *trnI* | N | 1-65 | 65 | 0 | GAT |  |  |
| *trnI* | N | 73-137 | 65 | 7 | GAT |  |  |
| *trnI* | N | 145-209 | 65 | 7 | GAT |  |  |
| *trnI* | N | 216-280 | 65 | 6 | GAT |  |  |
| *trnI* | N | 288-352 | 65 | 7 | GAT |  |  |
| AT-rich | J | 353-669 | 317 | 0 |  |  |  |
| *trnQ* | N | 670-738 | 69 | 0 | TTG |  |  |
| *trnM* | J | 738-801 | 64 | -1 | CAT |  |  |
| *ND2* | J | 802-1824 | 1023 | 0 |  | ATG | TAA |
| *trnW* | J | 1823-1890 | 68 | -2 | TCA |  |  |
| *trnC* | N | 1883-1944 | 62 | -8 | GCA |  |  |
| *trnY* | N | 1945-2007 | 63 | 0 | GTA |  |  |
| *COX1* | J | 2009-3544 | 1536 | 1 |  | CGA | TAA |
| *trnL2* | J | 3540-3603 | 64 | -5 | TAA |  |  |
| *COX2* | J | 3605-4292 | 688 | 1 |  | ATG | T |
| *trnK* | J | 4293-4360 | 68 | 0 | CTT |  |  |
| *trnD* | J | 4361-4426 | 66 | 0 | GTC |  |  |
| *ATP8* | J | 4427-4588 | 162 | 0 |  | ATC | TAA |
| *ATP6* | J | 4585-5259 | 675 | -4 |  | ATA | TAA |
| *COX3* | J | 5259-6045 | 787 | -1 |  | ATG | T |
| *trnG* | J | 6046-6107 | 62 | 0 | TCC |  |  |
| *ND3* | J | 6108-6461 | 354 | 0 |  | ATA | TAG |
| *trnA* | J | 6460-6525 | 66 | -2 | TGC |  |  |
| *trnR* | J | 6526-6585 | 60 | 0 | TCG |  |  |
| *trnN* | J | 6584-6647 | 64 | -2 | GTT |  |  |
| *trnS1* | J | 6645-6711 | 67 | -3 | GCT |  |  |
| *trnE* | J | 6861-6922 | 62 | 149 | TTC |  |  |
| *trnF* | N | 6923-6986 | 64 | -2 | GAA |  |  |
| *ND5* | N | 6987-8721 | 1735 | 0 |  | ATG | T |
| *trnH* | N | 8722-8784 | 64 | 0 | GTG |  |  |
| *ND4* | N | 8784-10,129 | 1346 | -1 |  | ATG | TA |
| *ND4L* | N | 10,123-10,419 | 297 | -7 |  | ATG | TAA |
| *trnT* | J | 10,426-10,488 | 63 | 6 | TGT |  |  |
| *trnP* | N | 10,489-10,553 | 65 | 0 | TGG |  |  |
| *ND6* | J | 10,556-11,074 | 519 | 2 |  | TTG | TAA |
| *CYTB* | J | 11,074-12,208 | 1135 | -1 |  | ATG | T |
| *trnS2* | J | 12,209-12,273 | 65 | 0 | TGA |  |  |
| *ND1* | N | 12,282-13,229 | 948 | 8 |  | ATT | TAA |
| *trnL1* | N | 13,230-13,293 | 64 | 0 | TAG |  |  |
| *rrnL* | N | 13,294-14,508 | 1215 | 0 |  |  |  |
| *trnV* | N | 14,509-14,577 | 69 | 0 | TAC |  |  |
| *rrnS* | N | 14,578-15,354 | 777 | 0 |  |  |  |
