# Supplementary material for: Mitochondrial Genome Characterization of Six Spiny Crawler Mayflies and Comparative Analysis Within Ephemerellidae (Ephemeroptera: Pannota)
Source: Ecol Evol. 2026 Jan 8;16(1):e72935. doi: 10.1002/ece3.72935 (PMC12782776; doi:10.1002/ece3.72935)
Supplement: Supplementary file 7 — Table S6: Annotation and gene organization of the Uracanthella punctisetae mitogenome. [file ECE3-16-e72935-s009.docx]

| **Gene** | **Strand** | **Nucleotide no.** | **Size(bp)** | **IN** | **Anticodon** | **Start codon** | **Stop codon** |
| --- | --- | --- | --- | --- | --- | --- | --- |
| *trnI* | N | 1-68 | 68 | 0 | GAT |  |  |
| AT-rich | J | 69-800 | 732 | 0 |  |  |  |
| *trnQ* | N | 801-869 | 69 | 0 | TTG |  |  |
| *trnM* | J | 869-933 | 65 | -1 | CAT |  |  |
| *ND2* | J | 934-1956 | 1023 | 0 |  | ATT | TAA |
| *trnW* | J | 1955-2023 | 69 | -2 | TCA |  |  |
| *trnC* | N | 2016-2079 | 64 | -8 | GCA |  |  |
| *trnY* | N | 2080-2146 | 67 | 0 | GTA |  |  |
| *COX1* | J | 2148-3683 | 1536 | 1 |  | CGA | TAA |
| *trnL2* | J | 3679-3743 | 65 | -5 | TAA |  |  |
| *COX2* | J | 3745-4432 | 688 | 1 |  | ATG | T |
| *trnK* | J | 4433-4502 | 70 | 0 | CTT |  |  |
| *trnD* | J | 4503-4568 | 66 | 0 | GTC |  |  |
| *ATP8* | J | 4569-4730 | 162 | 0 |  | ATC | TAA |
| *ATP6* | J | 4727-5401 | 675 | -4 |  | ATA | TAA |
| *COX3* | J | 5401-6189 | 789 | -1 |  | ATG | TAA |
| *trnG* | J | 6189-6250 | 62 | -1 | TCC |  |  |
| *ND3* | J | 6251-6604 | 354 | 0 |  | GTG | TAG |
| *trnA* | J | 6603-6667 | 65 | -2 | TGC |  |  |
| *trnR* | J | 6667-6728 | 62 | -1 | TCG |  |  |
| *trnN* | J | 6726-6790 | 65 | -3 | GTT |  |  |
| *trnS1* | J | 6788-6854 | 67 | -3 | GCT |  |  |
| *trnE* | J | 6855-6918 | 64 | 0 | TTC |  |  |
| *trnF* | N | 6917-6980 | 64 | -2 | GAA |  |  |
| *ND5* | N | 6982-8721 | 1740 | 1 |  | GTG | TAA |
| *trnH* | N | 8722-8786 | 65 | 0 | GTG |  |  |
| *ND4* | N | 8786-10,132 | 1347 | -1 |  | ATG | TAA |
| *ND4L* | N | 10,126-10,422 | 297 | -7 |  | ATG | TAA |
| *trnT* | J | 10,426-10,489 | 64 | 3 | TGT |  |  |
| *trnP* | N | 10,490-10,555 | 66 | 0 | TGG |  |  |
| *ND6* | J | 10,558-11,076 | 519 | 2 |  | TTG | TAA |
| *CYTB* | J | 11,077-12,213 | 1137 | 0 |  | ATG | TAG |
| *trnS2* | J | 12,217-12,276 | 60 | 3 | TGA |  |  |
| *ND1* | N | 12,301-13,239 | 939 | 24 |  | ATG | TAA |
| *trnL1* | N | 13,240-13,304 | 65 | 0 | TAG |  |  |
| *rrnL* | N | 13,305-14,528 | 1224 | 0 |  |  |  |
| *trnV* | N | 14,529-14,597 | 69 | 0 | TAC |  |  |
| *rrnS* | N | 14,598-15,435 | 838 | 0 |  |  |  |

**Table S6.** Annotation and gene organization of the *Uracanthella punctisetae* mitogenome.

Note: IN: Length of intergenic spacer, negative values indicate gene overlap.
