# Supplementary material for: Mitochondrial Genome Characterization of Six Spiny Crawler Mayflies and Comparative Analysis Within Ephemerellidae (Ephemeroptera: Pannota)
Source: Ecol Evol. 2026 Jan 8;16(1):e72935. doi: 10.1002/ece3.72935 (PMC12782776; doi:10.1002/ece3.72935)
Supplement: Supplementary file 8 — Table S7: A + T content (%) of different mitochondrial genomic regions in Ephemerellidae species. [file ECE3-16-e72935-s007.docx]

| **Species** | **mitDNA** | **PCG3** | **PCG12** | **PCG123** | **rRNA** | **tRNA** |
| --- | --- | --- | --- | --- | --- | --- |
| *Serratella zapekinae* | 65.16 | 71.59 | 61.16 | 64.64 | 68.51 | 66.64 |
| *Torleya grandiforceps* | 61.17 | 62.93 | 59.60 | 60.71 | 63.10 | 63.57 |
| *Torleya tumiforceps* | 62.65 | 67.72 | 59.92 | 62.52 | 64.28 | 64.53 |
| *Torleya mikhaili* | 64.45 | 68.29 | 60.75 | 63.26 | 66.83 | 67.30 |
| *Cincticostella fusca* | 60.32 | 60.27 | 58.89 | 59.35 | 64.07 | 63.87 |
| *Serratella ignita* | 64.67 | 71.37 | 60.40 | 64.06 | 67.25 | 66.20 |
| *Teloganopsis jinghongensis* | 66.53 | 75.81 | 61.45 | 66.24 | 68.59 | 68.21 |
| *Torleya nepalica* | 62.36 | 65.69 | 60.42 | 62.17 | 63.70 | 61.08 |
| *Drunella ishiyamana* | 64.82 | 70.82 | 61.22 | 64.42 | 68.27 | 66.48 |
| *Cincticostella gosei* | 60.83 | 62.24 | 58.36 | 59.65 | 64.59 | 64.15 |
| *Uracanthella punctisetae* | 65.05 | 70.51 | 60.41 | 63.77 | 66.97 | 69.26 |
| *Cincticostella femorata* | 60.58 | 61.92 | 58.81 | 59.85 | 64.49 | 64.23 |

**Table S7.** A+T content (%) of different mitochondrial genomic regions in Ephemerellidae species.
