# Supplementary material for: Mitochondrial Genome Characterization of Six Spiny Crawler Mayflies and Comparative Analysis Within Ephemerellidae (Ephemeroptera: Pannota)
Source: Ecol Evol. 2026 Jan 8;16(1):e72935. doi: 10.1002/ece3.72935 (PMC12782776; doi:10.1002/ece3.72935)
Supplement: Supplementary file 9 — Table S8: AT‐ and GC‐skew values of different mitochondrial genomic regions in six newly sequenced Ephemerellidae species. [file ECE3-16-e72935-s006.docx]

|  | **AT-skew** | | | | **GC-skew** | | | |
| --- | --- | --- | --- | --- | --- | --- | --- | --- |
|  | **mitDNA** | **PCG123** | **rRNA** | **tRNA** | **mitDNA** | **PCG123** | **rRNA** | **tRNA** |
| *Cincticostella femorata* | -0.0424 | -0.1796 | 0.0323 | 0.0254 | -0.1768 | -0.0093 | 0.2382 | 0.1111 |
| *Uracanthella punctisetae* | -0.0504 | -0.1975 | 0.0749 | 0.0292 | -0.1603 | -0.0452 | 0.2642 | 0.1267 |
| *Cincticostella gosei* | 0.0013 | -0.2006 | 0.0462 | 0.0125 | -0.1487 | -0.0178 | 0.2676 | 0.1667 |
| *Drunella ishiyamana* | -0.0552 | -0.1927 | 0.0675 | 0.0105 | -0.163 | -0.0091 | 0.2767 | 0.1172 |
| *Torleya nepalica* | -0.0406 | -0.2203 | 0.0558 | 0.024 | -0.1874 | 0.0114 | 0.2188 | 0.1377 |
| *Teloganopsis jinghongensis* | -0.0214 | -0.1953 | 0.0768 | -0.0185 | -0.1683 | -0.0254 | 0.279 | 0.115 |

**Table S8.** AT- and GC-skew values of different mitochondrial genomic regions in six newly sequenced Ephemerellidae species.
